# Design and performance analysis of a rectenna system for charging a mobile phone from ambient EM waves

**DOI:** 10.1016/j.heliyon.2023.e13964

**Published:** 2023-02-22

**Authors:** Pankaj Chandra Kar, Md. Ariful Islam

**Affiliations:** a Department of Information and Communication Technology, Comilla University, Cumilla, 3506, Bangladesh; b Department of Robotics and Mechatronics Engineering, University of Dhaka, Dhaka, 1000, Bangladesh

**Keywords:** Radio-frequency energy harvesting system (RF-EHS), Multiband antenna, Energy detection based spectrum sensing, Impedance matching network, Rectifier/voltage multiplier, Power management unit, Power conversion efficiency, Impact of mobility

## Abstract

Advances in information technology have dramatically enhanced mobile phones. Power capacity is one of the most significant limitations of a mobile phone. As a result, efficient energy management in such devices is critical everywhere. The goal of this research is to find a way to charge electronic devices wirelessly using radio frequency (RF) electromagnetic (EM) waves (Rectenna using energy detection-based spectrum sensing). Mechanical deformations cause frequency detuning, which lowers the effectiveness of antennas and rectennas that would otherwise allow wireless communication and RF energy harvesting in the far field. A rectenna based on a stretchable multiband antenna is designed as a self-powered system to perform reliably and integrate RF power received across its multiband despite mechanical deformations. Depending on what the battery needs, the proposed multiband antenna will work at 900 MHz, 1800 MHz, 2100 MHz, and 2.45 GHz as both an RF transducer and an RF energy harvester. Depending on the received RF power density (high), the receiving RF wave will be utilized for both communication and RF energy harvesting (RF-EH) when the battery's current voltage is less than 20% (referred to as “low voltage”). Otherwise, the received RF wave will be used only for RF-EH. The installed multiband rectifiers function perfectly in terms of efficiency and bandwidth. This proposed technique would reduce the charging crisis by 60–90% depending on the location of the mobile phone or receiver of ambient EM signals. This paper could help researchers in the field of RF energy-based wireless charging systems.

## 1 Introduction

Wireless devices have grown more popular in recent years in a number of applications, such as mobile phones and sensor networks, due to their reliable and consistent communication. Charging batteries has become more important and reliable as wireless applications have grown in popularity. Charging batteries is still a big problem for any wireless sensor node that is in a place where things change often. Things get worse when there are many devices spread out over a large area or in places that are hard to find. To harvest energy, existing electromagnetic signals from ambient radiative components, such as cell towers, can be used [[1], [2], [3], [4], [5]]

In the late 1950s, a plan to power a helicopter with microwaves started the story of RF power harvesting in space [6,7]. Hertz proved the presence of EM waves experimentally in 1888 [8]. An oscillating electric charge generates EM waves, which is the basis for this experiment [9]. The energy for these waves is provided by the kinetic energy of the oscillating charge [10]. When inductive charging systems work at a low frequency, the radiation from the system can be ignored, and lumped circuit parts can be used to approximate the antennas [11]. Two inductors, which are also known as coils, can be used as a receiving and sending antenna to do magnetic induction. In Ref. [12], authors found that this is because coils can make strong magnetic fields at the edge that is closest to them.

Many other conductive materials, such as Ag-PDMS composite [13] and metal nanowires [14,15] have been investigated for use in manufacturing the antennas. Due to the poor conductivity of stretchable and flexible conductive materials, CNT composite-based deformable antennas were developed to overcome the challenges [16,17]. On an elastomeric substrate, stretchable metal antennas are fabricated by combining laser-induced graphene (LIG) patterns with a selective metal surface coating [18]. Also, the all-LIG electronics could open up new diagnostic monitoring and treatment options by combining different LIG sensors with wireless transmission and energy harvesting modules [19]. Based on carbon nanotube (CNT) sheets on polydimethylsiloxane (PDMS), Lipomi and colleagues designed a pressure and strain sensor with a skin-like structure [20,21]. Stretchable multiband antennas and rectennas could be used for things like self-powered devices, remote environmental monitoring, and renewable energy [22].

### 1.1 RF-EH techniques

The RF-EH is different from other ways to get energy, like the wind, the sun, and vibrations, in the following ways: (a) The ability to control and transfer energy continuously over a distant location; (b) The RF-RH fluctuates significantly depending on where a network node is located because the overall RF-EH is related to the distance between the dedicated RF source and the ambient RF source; (c) In an RF-EHN configuration, the gathered energy is somewhat predictable and reliable for long-term operation over a specified distance.

#### 1.1.1 RF-EH from dedicated RF sources

Low power values can be used to extract energy from the environment, while high power values can be used to transport energy. A device that extracts RF energy from a dedicated source at a relatively close distance is anticipated to provide power levels in the range of 50 nW/cm^2^. An RFID (radio frequency identification) chip is an example of anything that can be powered by an RFID reader. Devices that are embedded are able to have their batteries recharged because of the dedicated power source [23].

#### 1.1.2 RF-EH from ambient RF sources

Modulating the signal (for example, by changing the frequency and power) transmits the proper amount of electricity to the sensing device. In spite of the fact that they are transmitters of stable power, static sources are not simple. As background noise, one might hear broadcast radio, mobile base stations, or television. These are all possible sources. Note that dynamic sources are not discussed in this paper.

**Fig. 1:**
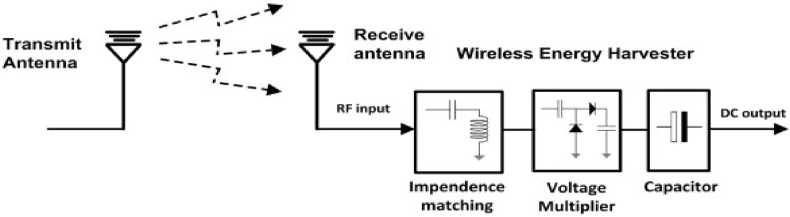
Conceptual block diagram of RF-EH System.

Fig. 1 shows how to collect RF energy from the surrounding environment. A matching network with inductive and capacitive parts makes sure that the antenna sends as much power as possible to the voltage multiplier. The voltage multiplier converts the RF power into DC power and multiplies the resulting voltage. When there isn't enough or any energy coming from the outside, the energy storage acts as a backup and makes sure the load gets power smoothly.

**Fig. 2:**
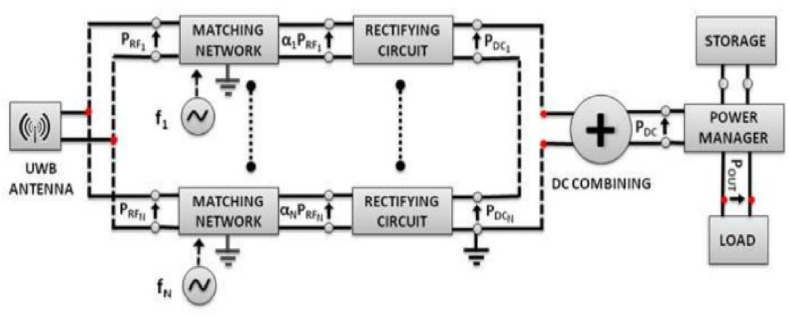
An architecture of multi-band energy harvesting system.

**Fig. 3:**
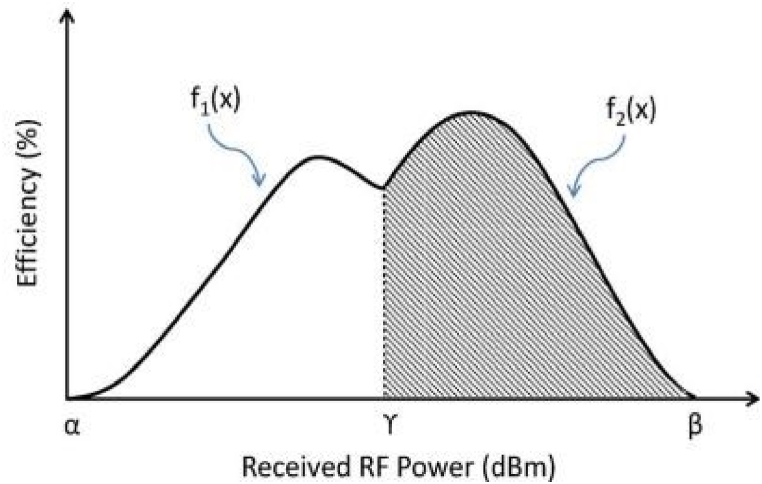
The efficiency curves of two sister circuits for energy harvesting, one for low power dissipation and one for high power dissipation [30].

Fig. 2 shows how an RF harvesting system is put together and what factors affect its overall performance. A source, impedance matching circuits, antennas that send or receive signals, a voltage multiplier, and a capacitor are all needed for an antenna system. An antenna picks up RF energy from the environment. Then, this energy is fed into a frequency-selective circuit in order to reduce transmission loss and boost the input voltage feeding into the rectifier circuit to make it as efficient as possible. The matching network generates AC (alternating current) voltage at its output, which must be converted to DC voltage by a special power converter. On the other hand, the DC voltage that is generated by the rectifier circuit is incredibly low and cannot be used to power circuits directly. By adding a DC-DC boost circuit, the voltage made by the RF-DC rectifier has been raised. The output voltage is then governed using a regulator circuit. This energy is recorded and stored in a capacitor or battery for subsequent use [[24], [25], [26], [27], [28], [29]] (see Fig. 3 [30]).

#### 1.1.3 Transmission of radio frequency energy between mobile devices

Wireless communication devices can work as RF sources, which lets them reliably transfer power between devices that are close to each other. In the past, some RF energy transmitters and receivers were made that could send and receive both power and information at the same time. In Ref. [31], authors say that mobile devices that can send RF energy based on information and destined for relay nodes can be used to stop the imbalanced use of energy.

#### 1.1.4 Utilization of RF energy in remote areas

Radio-frequency (RF) signals gathered by antennas from electromagnetic radiation travel several kilometers before rectifier circuits (far-field RF-EHS) convert them to electricity [32]. The term “far-field RF-EH” refers to both the ambient far-field RF-EH as well as the dedicated far-field RF-EH [33]. Practically, the propagation characteristics of the environment will have a significant impact on the amount of received power [34]. Some of the negatives of using RF-H are that it might result in energy dissipation, path loss, fading, and shadowing [35]. The Friis transmission and the equivalent isotropically radiated power (EIRP) are two significant upper limits for the RF-EHS. First, the available power at the antenna should never be more than the EIRP, no matter how far away the signal is. Second, even when the signal is clearly reflected, the signal's power falls practically to the level of the Friis transmission. In the study, path loss is taken into account to indicate the signal strength in the far-field region.

## 2 Literature review

The double circular polarization patch antenna was made by authors in Ref. [36]. They used high-order harmonic refusal to make it. The suggested structure can gather RF signals in all polarization modes at 2.45 GHz. The author made a dual circularly polarized framework with six bands by streamlining the process. This antenna can grasp frequency bands between 470 MHz and 2.5 GHz. In Ref. [37], Digital TV, cell phones, and Wi-Fi all use these ranges of frequencies. The authors [38] of the study, developed a multiband antenna for the frequency bands 2.61 GHz and 7.1 GHz. This frequency band has been proposed for the subsequent generation of wireless communication technologies. The planned antenna has a bandwidth that can accommodate transmissions of 300 MHz, 490 MHz, 2.61 GHz, and 7.1 GHz, respectively. The authors in Ref. [11] suggest a portable rectenna for use in the ultra-high frequency range. An adhesive conductive fabric is used to create the antenna construction on a pile and jean substrate. The back of the antenna is connected to the full-wave bridge rectifier using a layer of denim. An antenna that was designed by authors [39] can be utilized for a variety of wireless applications, including wireless fidelity (Wi-Fi), Wi-Max, and 3G, all while operating within the microwave frequency bands of L, S, C, and X in the electromagnetic spectrum. The study looked into whether it would be possible to get around the matching network block by using an off-center-fed dipole antenna. With all this antenna design, a high input impedance that can be straightforwardly adapted to the rectenna system can be achieved [40]. In Ref. [25], authors said that for a wide variety of DC loads, the conversion efficiency should be maintained using resistance compaction networks. When antennas are put on top of solar panels, it is easier to collect energy [41]. At night, the antennas are the only form of energy collection. This integration enables the consistent harvesting of energy from two sources. In Refs. [42,43] authors describe a coplanar Vivaldi antenna structure with a solar panel built into it. On an FR-4 substrate, a microstrip transmission line is printed. This helps the solar panel and keeps the antenna fed. In Ref. [44], authors also looked into a reversed antenna that could be printed on an FR-4 substrate and attached to a solar cell. Conventional mechanical sensors are limited to sensing only one deformation mode, such as uniaxial strain pressure or torsion, and can only tolerate deformations of up to 5%. When deposited as thin films, nanomaterials such as metal nanoparticles, metal nanowires, carbon nanotubes, and graphene-based metals are flexible. The author developed a mechanical sensor by embedding low-strength, wetspun SWCNTs wire in PDMS. It is extremely sensitive to stretching, bending, and twisting [45]. In recent years, numerous types of stretchable antennas suitable for wearable system applications have been described [46]. The sensor and antenna are typically treated as two distinct components in the existing literature [47]. There are only a few instance where these features were merged and utilized as single device [48,49,[50], [51], [52]]. Microstrip patch antennas are used to reduce the amount of EM energy dissipated by the human body. It is also capable of strain detection. It is also capable of detecting strain, which involves monitoring the change in resonance frequency to determine the amount of deformation [53]. The authors found that a 15% maximum tensile strain in the feeding direction of a stretchable microstrip antenna can result in a decrease in the resonant frequency [16]. In Ref. [11], authors study the modeling and analysis of antennas to achieve greater levels of operational effectiveness in electricity transmission. Experiments and computer simulations use the circuit hypothesis and full wave electromagnetic assessment to look at the relationship between how well wireless power transfer works and how antennas are made. This is needed so that antennas can be made that work well for highly effective wireless charging. The efficiency of a Class-F rectifier during RF-to-dc conversion is represented mathematically in Ref. [40]. Concerning the time diode currents and voltages, this model gives an explanation for each type of diode power loss that happens in the rectifier because of the breakdown voltage, diode series resistor, junction capacitor, and built-in potential. In Ref. [25], authors came up with a new rectifier booster regulator (RBR) that can change AC energy into DC energy and boost the high voltage output. Blending a Greinacher rectifier with two Dickson charge pumps results in a full-wave rectifier. In Ref. [41], authors made a unique microwave rectifier with two diodes and microstrip access that works at 2.45 GHz. This circuit was made by using both EM and circuit techniques as part of a global analysis. Two rectifiers with different Schottky diodes (HSMS 2860 and HSMS 2820) were built and measured on an Arlon 25 N substrate. In Ref. [54], authors described the construction and function of a novel antenna that works on three frequency bands to capture RF energy from 900 MHz to 1900 MHz cellular network frequency bands and 2.4 GHz Wi-Fi sources. The authors of [55], designed a theoretical antenna and circuit to harvest energy from nearby EM waves and power a portable electronic device. A multiplier circuit was formed in this work. For an RF/DC energy conversion system to be effective, it must be able to harness extremely weak RF signals from a great distance. The passive rectifier circuits were optimized for the far field [56]. The circuit is located 44 m away from a 4 kW EIRP source. The simulation results showed that L and T matching types are preferable to Pi-matching types for a 2-level Diskon Voltage Multiplier (DVM), but for a 6-stage DVM, Pi-matching is just as good as T and L matching types when the HSMS-2852 diode modal is used [57].

The significant contributions to this study are summarized below.

• Firstly, to accumulate more power than a single-band antenna (for microwave and mm-wave applications), we intend to investigate various design concerns and measurement techniques for a stretchy and flexible antenna (a reconfigurable multiband patch antenna).
• To reduce polarization mismatch loss, the proposed single-band antennas are fed differentially (dual polarization).
• Third, between the antenna and the energy-based spectrum sensing network is a band pass filter (Butterworth) that uses a Pi-matching network to get rid of higher-order harmonics that reduce the system's efficiency. A series-LC resonating circuit is used for a single-band antenna instead of a band-pass filter.
• Fourthly, two sister N-stage voltage multiplier/rectifier circuits using Schottky diodes are designed. To enhance the effectiveness of the rectifier network, energy detection-based spectrum sensing is used. Depending on the energy detector's output, the HSMS-2822 (for LPD) or HSMS-2852 (for LPD)-based network will be active at a certain time.
• Fifth, an inductor is added to the DVM to store energy in the magnetic field during the negative cycle and release it during the positive cycle. This makes sure that the power to the load is smooth. A low-pass filter is also used in each sister network to cut down on voltage ripple.
• Sixth, depending on what the cell phone battery needs, two super capacitors, a smart switching system, a voltage regulator, and an auto cutoff circuit are put together to make a power management unit. To boost the output voltage, multiple RF/DC converters are connected in series configuration.
• In this paper, the consequences of motion on mobile phone charging time and dissipated power have been analyzed.
• During the design phase of the RF harvesting system, internal state of charge (SoC) resistance has also been taken into consideration.

## 3 Evaluation metrics of wireless power harvesting system

The value of the evaluation varies with the nature of the application being evaluated. However, significant parametric properties, such as sensitivity, efficiency, output power, and operating distance, serve as the fundamental evaluation metrics for comparison analysis [58,59].

### 3.1 RF input power

RF-EH circuit itself is nonlinear since diodes are nonlinear electronic components. This means that the impedance of the energy-harvesting circuit changes depending on how much power the antenna transmits. It is the preferred method to match the impedances of the receiver circuit and antenna at a fixed input power level. This makes us want to make two LDP and HDP circuits that are very different from each other and have their own constant impedances. The crossover point, where the LPD and HPD efficiency curves connect, splits the desired range of −20 to 20 dBm in half. The overall efficiency can be calculated by combining the two curves that lie on either side of the junction point.

### 3.2 Frequency range and distance

One of the most crucial fundamental functions for RF power is the operating range, or distance, between the transmitter (Tx) and receiver (Rx). This is related to the operating frequency of the system. Since higher frequency signals have longer wave lengths while traveling over wireless media, they are more susceptible to attenuation than lower-frequency signals. Compared to their counterparts, low-frequency signals can penetrate matter more deeply.

### 3.3 Resonance factor

Resonance happens when a system can easily store energy in one form and move it to another form, like kinetic or potential energy. For maximum output power, most energy harvesters are designed to operate at resonant frequencies. A system can oscillate on its own or be forced to do so at its resonance frequency. Increasing the size of the antenna decreases its resonance frequency. The resonance factor is correlated with resonance intensity and bandwidth (also known as the Q factor). The narrow resonant bandwidth combined with a strong voltage gain is referred to as a high Q factor. Q factor expression is derived as follows [60]:

(1) $$Q=2\pi \frac{E_{stored}}{E_{dissipated}}$$

where $E_{stored}$ is the sum of all energy that has been stored and $E_{dissipated}$ represents the quantity of energy that is lost during each cycle.

### 3.4 Output power

Another important way to measure how well a power harvesting system works is by its output power, which is often called “DC power” and is what happens when the voltage and current of the load are added together. By measuring the load voltage, you can see how well a system works when it is dependent on the impedance of the load. For instance, it is more important to have a high voltage (V) than a high current (I) when the application is a sensor. On the other hand, I predominates over V in applications using LEDs or electrolysis.

### 3.5 Efficiency of power conversion

The effectiveness of a rectifier circuit can be determined by comparing the RF power it receives with the DC power it outputs to the load. The following is the mathematical demonstration of the point [61]:

(2) $$\eta_{PCE}=\frac{P_{Load}}{P_{retrived}}$$

where $P_{Load}$ indicates the amount of electricity that is being provided to the load, while $P_{retrived}$ is the power that is derived from the antenna itself. It is crucial to notice that the expression does not account for RF transmission loss in space. How well a rectifier circuit converts energy is a big part of how quickly it can take in energy. Because of this, an efficiency of close to 100% is necessary, which indicates that all of the energy that is fed into the rectifier needs to be transformed into direct current energy. It is crucial to precisely tune the impedance matching network and enhance the output load resistance.

The effectiveness of an RF-EH system is determined by the following elements: (a) The main things that go into making a receiving antenna are its efficiency and gain; (b) Maximum power transfer is ensured through impedance matching; (c) Power effectiveness of the rectifier circuit.

### 3.6 Sensitivity

The sensitivity is found by figuring out how much power the system needs to start up with the least amount of power. The results are shown below [61]:

(3) $$Sensitivity\;\left(dBm\right){=\log}_{10}\left(\frac{P}{1\;mW}\right)$$

where P is the least amount of power that the system needs to reach any desired outcome. Using impedance-matching networks can make the RF-DC rectifier circuit more sensitive. Increasing the sensitivity of regulatory functions by utilizing a DC/DC converter makes it easier to make changes in a wide range of RF energy harvesting applications.

### 3.7 Peak passive voltage

To obtain the passive peak voltage, one can use any one of three types of rectifiers: A bridge rectifier, a rectifier with a single diode, or an ideal diode rectifier. Negative voltages from the input signal are eliminated using diodes. By adding a capacitor to the rectifier circuit, the ripples in the DC output caused by the rectification process are smoothed out. This helps ensure that the DC output is continuous and uninterrupted. The relationship between $V_{peak}$ and $V_{TH}$ at the input determines the sensitivity and efficiency of the rectifier. $V_{peak}$ and the rectifier output voltage are correlated since the output voltage depends on how many stages there are in the rectifier ladder. By coupling the reactive and resistive components, passive voltage boosting may be achieved, as shown in Equation (4) [58];

(4) $$\frac{V_{rec}}{V_{ant}}=\frac{R_{rec}+{jX}_{rec}}{\left(R_{ant}+R_{rec}\right)+j\left(X_{ant}-X_{rec}\right)}$$

where $V_{rec}$: the input voltage to the rectifier. $V_{ant}$: the source of voltage for the antenna. $R_{ant}$: the resistor of the antenna. $X_{ant}$: the inductor of the antenna.

### 3.8 Dropout voltage regulator

A switching regulator, a linear regulator, and control logic make up the dropout voltage regulator. These three components work together to form the dropout voltage regulator. It is possible for voltage regulators, such as linear low dropout (LDO) regulators, to maintain the output voltage at the same level even if the load current or supply voltage is altered. Even if the load current or supply voltage fluctuates, the second must keep the proper output voltage. They outperform switching converters in terms of noise isolation. The LDO regulator is used by several portable battery-powered devices. Because of the dropout voltage $V_{DO}$ = ($V_{IN}$ − $V_{OUT}$), the power efficiency of LDOs is fundamentally limited. Equation (5) is used to determine how the regulation should be carried out [58].

(5) $${Regulator\;Dropout\;Voltage=V}_{dropout}{=(V}_{out}{-V}_{DD})$$

## 4 Architecture of RF-EH circuit

RF energy is consistently found in the environment, but most of the time it is not very strong. In fact, the small amount of radio frequency power means that the RF/DC converter circuit is not very good at converting power. Because each part of the RF-EHS has different losses and limits, it is hard to use this converted energy. A standard RF-EHS consists of an antenna, a matching network, a power conversion module, and a load to assure the system's feasibility. These parts are meant to make sure the system works. The architecture of the rectenna system included an antenna, a filter network, an impedance matching network, and a rectifier circuit. The antenna detects and gathers the RF energy, which the rectifier subsequently transforms into a DC signal. The “pre-rectifier”, often referred to as an RF input filter, decreases the amount of harmonics generated by the rectifier's presence of a nonlinear component. To ensure maximum power is transferred from the filter network to the rectifier circuit, an impedance matching network is inserted in between them.

### 4.1 Antenna design and considerations

An integral part of an RF-EHS is the antenna, which is mainly responsible for gathering RF energy from transmitters and delivering it to the recctenna system so that it can be rectified into DC voltage and used to power devices. This is one of the key functions of an RF power harvester. To transmit power as effectively as possible, antenna design is critical. The antenna's design poses a number of problems, most of which can be put under the headings of conversion efficiency, form factor, and bandwidth. This section will talk about some of the most important things to think about when designing something.

#### 4.1.1 Antenna characteristics

It is critical to remember that the frequency of radiation is inversely proportional to antenna size and a trade-off exists between them. Antenna designers need to use a design method that can be optimized to improve antenna performance while at the same time reducing antenna size. When making an antenna, the bandwidth, beamwidth, efficiency, and output gain are the most important things to think about.

The following is an explanation of how to describe the Q-factor of an antenna [59]:

(6) $$Q=\frac{CF}{BW}$$

where CF represents the center frequency and BW represents the bandwidth. Conversely, a narrow bandwidth is required to attain a higher Q-factor value. On the other hand, if you make the antenna smaller, Q goes down and bandwidth goes up. Using materials with a high dielectric constant is the simplest way to solve this problem. However, there are a few significant drawbacks to this strategy, including a narrow bandwidth and low gain due to surface wave excitation.

#### 4.1.2 Polarization

The direction in which an antenna's electromagnetic fields send energy away from the antenna is called its polarization. The polarization of an antenna in the E-plane is determined by the direction of the electromagnetic radiation it sends or receives. An antenna's polarization can be broken down into two main types: linearly (horizontally and vertically) or circularly. As a result, the power conversion efficiency is enhanced by a reduced amount of polarization mismatch loss. Even though circular polarization is good at converting, its main flaw is that it has a narrow bandwidth.

#### 4.1.3 Harmonic rejections

The energy harvesting circuit is nonlinear because it has diodes, which are electronic parts that are not linear. The performance of the entire circuit degrades as a result of electromagnetic interference caused by these harmonics that re-radiate into the transmitting antenna as well as the receiving antenna's related circuit. In order to resolve this issue, low-pass filters will need to be installed in the path that connects the antenna and the rectification circuit. For the objectives of high power radio frequency energy harvesting, rejection of harmonics is more important than ever before because of the increased severity of harmonics in the high-power spectrum.

#### 4.1.4 Reconfigurability

A regulated and reversible method by which an antenna may alter both its frequency and the manner in which it sends out waves is referred to as a reconfigurable antenna. By modifying antenna's construction, it may be tuned to a certain set of frequencies, which affects its physical properties in a way that simultaneously increases its bandwidth and decreases its size.

Changes to the way waves move through the transmission line can be used to control the difference in phase between the components that are radiating. The main features of the reconfigurable antenna are its ability to do more than one thing, its good isolation, its small size, its ability to block out-of-band signals, and the fact that it doesn't need a filtering element. All of these characteristics are present even without the presence of a filtering element. Reconfigurable antennas can be classified by their dynamically altered operational features, such as frequency, radiation pattern, or polarization.

*Frequency Reconfigurable Antennas:* Both electrical and mechanical methods can be used to fabricate such antennas. The electrical mechanism employs two types of tuning strategies: discrete and continuous. Radio frequency (RF) switches and varactor diodes can be utilized to produce discrete and continuous tuning, respectively. The mechanical mechanism employs tunable impedance loading components, such as liquid crystals and a metasurface, to accomplish frequency reconfiguration.

*Pattern Reconfigurable Antennas:* To modify the radiation pattern over a sphere, these antennas use rotatable or switchable components, such as metasurfaces or reactively loaded capacitive elements.

*Polarization Reconfigurable Antennas:* The above antennas are capable of tuning between linear polarizations, such as left-hand circular polarization (LHCP) and right-hand circular polarization (RHCP), due to the use of a multi-modal structure or metasurface (RHCP). In mobile networks, it is crucial to toggle among horizontal, vertical, and circular polarizations to cut down on polarization mismatch losses.

*Compound Reconfigurable Antennas:* Utilizing a parasitic pixel layer, these antennas simultaneously tune many antenna characteristics, such as frequency and radiation pattern, allowing individual adjustment of frequency response, radiation pattern, and polarization.

#### 4.1.5 Flexibility

A flexible antenna must have mechanical stability, efficiency, bandwidth, and radiative properties. Due to the antenna's expected bending and rolling in wearable or flexible energy harvesters, three tests are needed to verify its functionality [62].

**Fig. 4:**
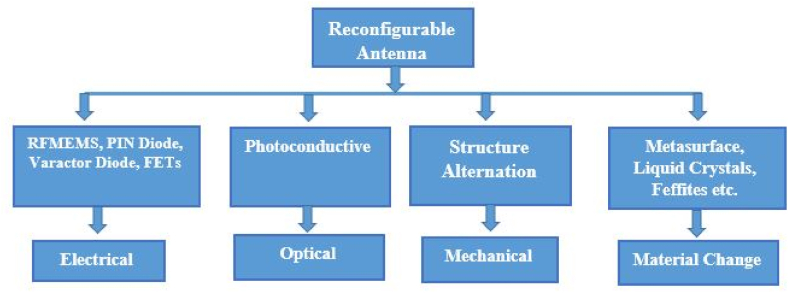
Antenna reconfiguration techniques [63].

• Analyzing the injected conductive ink for deformations and discontinuities and ensuring there are no fractures, wrinkles, or permanent folds necessitates a robustness and durability analysis under bending, rolling, and twisting.
• To comprehend how bending impacts on the resonant frequency and return loss, we must examine impedance mismatch.
• To ensure that the antenna's radiation pattern and gain are not distorted or otherwise diminished after being shaped to fit a curved surface, they must be subjected to rigorous testing (see Fig. 4 [63]).

**Fig. 5:**
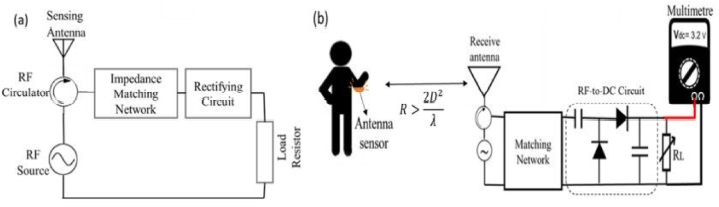
RF energy harvesting system (a) sensing antenna integrated into a rectenna (b) monitoring configuration utilizing a flexible antenna to transform the angular deflection of a human arm into a direct current voltage [64].

Depending on how the body moves, the angle of the bend decreases the resonant frequency. The schematic diagram of the RF/DC network of the proposed sensing antenna is depicted in Fig. 5(a). This output voltage has the potential to drive an actuator or sensor. Fig. 5(b) depicts a monitoring configuration utilizing a flexible antenna to transform the angular deflection of a human arm into a direct current voltage.

#### 4.1.6 Stretchability

**Fig. 6:**
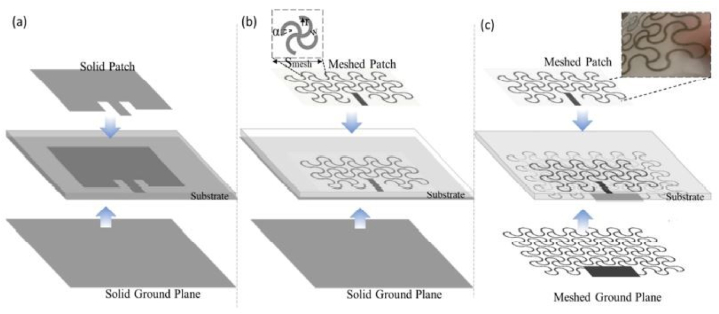
Three variations of the planned textile microstrip antennas are depicted in a schematic. (a) solid metal-plated microstrip patch antenna. (b) Convert the solid patch to a mesh pattern to enhance its stretchability. (c) Altering both the solid patch and the ground plane with mesh structures [64].

**Fig. 7:**
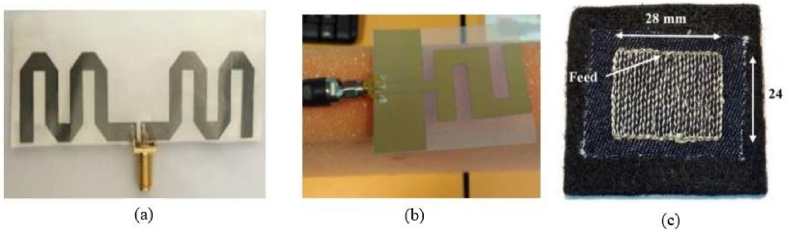
Different types of fabrication methods. (a) Meandered-line screen-printing antenna [67] (b) Inkjet-printed Z-shaped antenna [68] (c) Embroidered NMPA on a flexible felt substrate [69].

The performance of a RFEH system deteriorates due to the detuning of its resonant frequency caused by mechanical deformation in the far field. Stretchable wideband antennas and rectennas are designed to perform robustly and integrate received RF power across their entire bandwidth despite mechanical deformations. Stretchable wideband or multiband antennas and rectennas give prospects for applications such as self-powered devices, remote environmental monitoring, and renewable energy. For the radiation elements, traditional metals with stretchy structures (e.g., a serpentine or mesh geometry) can be investigated to improve the radiation efficiency and electromagnetic characteristics [65]. Since the resonance frequency shifts in response to tensile strain, the designed antennas may have other applications outside wireless communications as strain sensors. Fig. 6(a), (b), (c) depict that the antenna is non-stretchable, 40% stretchable and 100% stretchable respectively [66] (see Fig. 7 [75][76][77]).

#### 4.1.7 Material choice and fabrication methods

Electrical and physical qualities are typically associated with the antenna minimization technique. It can be classified by either topological or material approaches, where each category has a great deal of specific variety. The electromagnetic bandgap (EBG) structure reduces antenna size without affecting radiation efficiency. The conductive material is selected based on its electrical properties, whereas the material of the substrate is determined by its dielectric characteristics, sensitivity to mechanical deformations such as bending, wrapping, and twisting, resistance to the ambient environment, and susceptibility to miniaturization.

**Conductive Materials:** In order to maximize antenna efficiency, it is important to analyze materials that have a high electrical conductivity. Materials that conduct electricity should have low resistance and a high tolerance for damage from mechanical deformations. Several stretchable conductive materials boost their conductivity through doping, allowing mechanical strain and deformation to be applied with no reduction in antenna performance or efficiency.

**Substrate:** The compactness of an antenna relies on the dielectric constant of the substrate. While designing an antenna, one must balance the antenna's efficiency against its physical size. Substrates with durability, washability, flexibility, and stretchability are typically used in flexible antenna applications.

*Relative constant (Dielectric constant):* The dielectric constant influences the capacity to transfer varying signals over a fabric transmission line. It relies on various parameters, including temperature, frequency, surface texture roughness, purity, moisture content, and material homogeneity. As a result, the antenna's impedance bandwidth is improved, and its spatial waves are amplified, when the dielectric constant is reduced.

*Thickness of the dielectric materials:* The efficiency and bandwidth of an antenna are related to the thickness and dielectric constant of its substrate. It enables the prediction of input impedance, bandwidth, and resonance frequency. It also effects the antenna's geometry. The thin substrates are utilized to fabricate small-size antennas. Conversely, the thick substrates are used to fabricate large antenna patches [70,71].

*Surface electrical resistance of conductive materials:* The performance of a flexible antenna is greatly affected by the conductive material used for its patch and ground. The surface resistivity is found by dividing the DC voltage lost by the surface current per unit length. Dielectric fabrics are exceedingly flexible and elastic, and they conform to the shape of a human body in a wide variety of ways (including bending, twisting, elongating, and curving).

**Fabrication Methods:** It is vital to adopt an appropriate fabrication approach in order for the flexible antenna to offer superior stability and electrical performance. Screen-printing, Ink Jet Printing, Chemical Etching, sewing, and embroidery are among the most often employed fabrication methods [72,73]. Screen printing is a fast, affordable, and highly efficient method for fabricating the antenna [74]. Among the many low-cost printing technologies, inkjet printing is notable for its ability to create extremely precise patterns at minimal cost [75,76]. Without wasting any ink, it sprays a single droplet of ink from the nozzle [77]. Sewing and embroidery do not utilize an epoxy substance on the fabric, which could alter its electrical properties. This geometry is far more elastic than metallic antennas, making it a preferred solution to conventional antennas in device applications [78]. The use of LIG-based antennas reveals the feasibility of constructing a small, simple-to-fabricate, sensitive, and flexible antenna-based Internet of Things (IoT) sensor for use in motion detection, structural health monitoring, and industrial strain sensing [79]. Ablation sensitivity is a function of input power, pulse width, and scanning rate [80]. Different types of fabrication methods are shown in bellow.

### 4.2 Energy sensitivity

The most challenging aspect of designing an RF-DC converter is achieving a high DC output voltage with a low RF input power. Most of the time, energy detection is used as part of the sensing process for the optimization framework that figures out when the LPD/HPD subcircuit switches over. In the range of −20 to 20 dBm, it enhances the efficiency of energy harvesting modules, such as the gain of the voltage multiplier, by defining which sister circuit will be active depending on its threshold at which an energy collection system becomes functional.

### 4.3 Impedance matching network

**Fig. 8:**
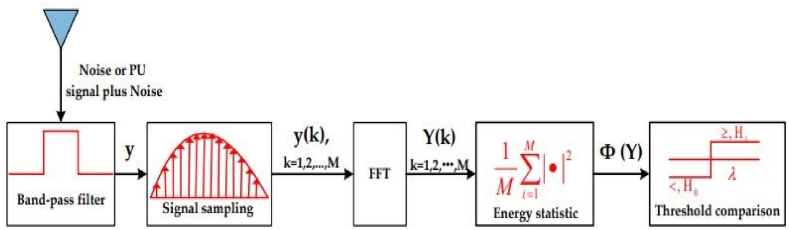
Structure of an energy sensitivity system [83].

When compared to a narrow-band rectenna, the output voltage of a multiband, broadband, or rectenna array may be higher. For RFEHS, a multiband antenna can be used to capture different RF waves. Quad band antennas, for example, are widely available on the market and typically operate at 900 MHz/1800 MHz/1900 MHz/2.4 GHz. The energy harvesting circuit is nonlinear because it has diodes, which are electronic parts that are not linear. This means that the impedance of the energy-harvesting network changes based on how much power the antenna sends. The antenna may be tuned to its resonant frequency using an LC matching circuit. A matching network of inductive and capacitive parts makes sure that a 50-Ω antenna gets the most power at its resonant frequency, which the voltage multiplier turns into DC power. When analyzing the voltage multiplier at high frequencies, each capacitance must be treated as a short circuit, and each diode must be parallel or anti-parallel to the input [81]. Consequently, upon entering the voltage multiplier, the capacitances of all diodes are parallel. When designing an LC power matching network, the parameters L and C are typically chosen as: L = $\frac{QR_{A}}{\omega_{0}}$ and C = $\frac{Q}{{R_{eq}\;\omega }_{0}}$ [82]. In this expression, $R_{A}$ represents the antenna resistance and $R_{eq}$ represents the voltage multiplier's equivalent input resistance. The LC network's quality factor is denoted by the parameter Q. Q = $\sqrt{\frac{R_{eq}}{R_{A}}-1}$. A parallel inductance L can compensate the voltage multiplier's equivalent input capacitance. At the operating frequency, which fluctuates with the voltage across the diodes, the value of the inductance, L, is chosen to resonate with the average value of the input capacitance. The variations of the input impedance lead to the condition, ΔC= $\frac{Q}{2\pi f_{0}}$ [81]. In a voltage multiplier, ΔC represents the largest deviation of the input capacitance from its average value (see Fig. 8 [83]).

**Fig. 9:**
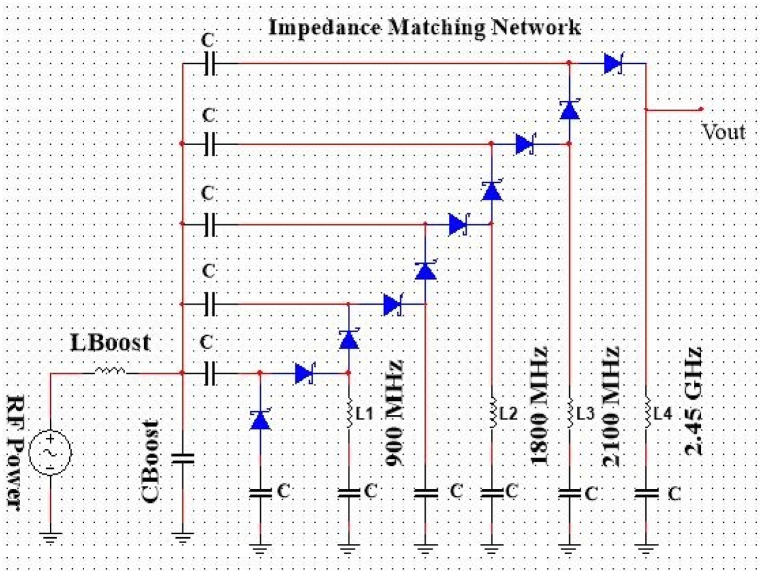
Schematic circuit diagram of multiband impedance matching network.

Fig. 9 depicts the component values and schematic representation of the rectifier network. The Schottky diode from Avago Technologies is suitable for a high-sensitivity system. A multi-stage rectifier was selected for its excellent sensitivity as well as the simplicity of its multi-band matching. Component values of the impedance matching network: $C_{Boost}$ = 2.2 pF, C = 0.2 pF, $L_{Boost}$ = 3 nH, *L*_1_ = 39 nH, *L*_2_ = 10 nH, *L*_3_ = 10 nH, *L*_4_ = 10 nH. The proposed antenna has an output impedance equal to the input impedance of the rectifier network, which is 50 Ω; hence, a simple LC voltage boosting network with four lump inductors, *L*_1_, *L*_2_, *L*_3_, and *L*_4_, is utilized. The proposed rectifier will operate only at 900 MHz when all the inductors except *L*_1_ are short-circuited. Similarly, if all the other inductors except *L*_2_ are short-circuited then it will operate at 1800 MHz. Consequently, it is true for L3 and L4, which operate at 2100 MHz and 2.45 GHz, respectively. The rectifier has three matching frequencies in the 900 MHz, 1800 MHz, 2100 MHz bands when only *L*_4_ is shorted. When L_3_ is shorted, the matching at 2100 MHz is disabled, and only 900 MHz, 1800 MHz, 2.45 GHz are matched. If L_3_ and *L*_2_ are shorted, the matching at 1800 MHz, 900 MHz are off. It will work at all frequencies, depending on what the system needs. In this case, no inductor will short out.

### 4.4 Rectifiers or voltage multiplier design

In order to transform received signals into DC signals for the purpose of RF energy harvesting, voltage multipliers are utilized. RF energy harvesting circuits can be classified into two primary categories: Villard voltage doublers, commonly referred as Cockcroft-Walton voltage multipliers, and Dickson voltage multipliers. Both of these forms of voltage multipliers are known as Cockcroft-Walton voltage multipliers. Both Villard and Dickson topologies show no appreciable performance differences [84].

**Fig. 10:**
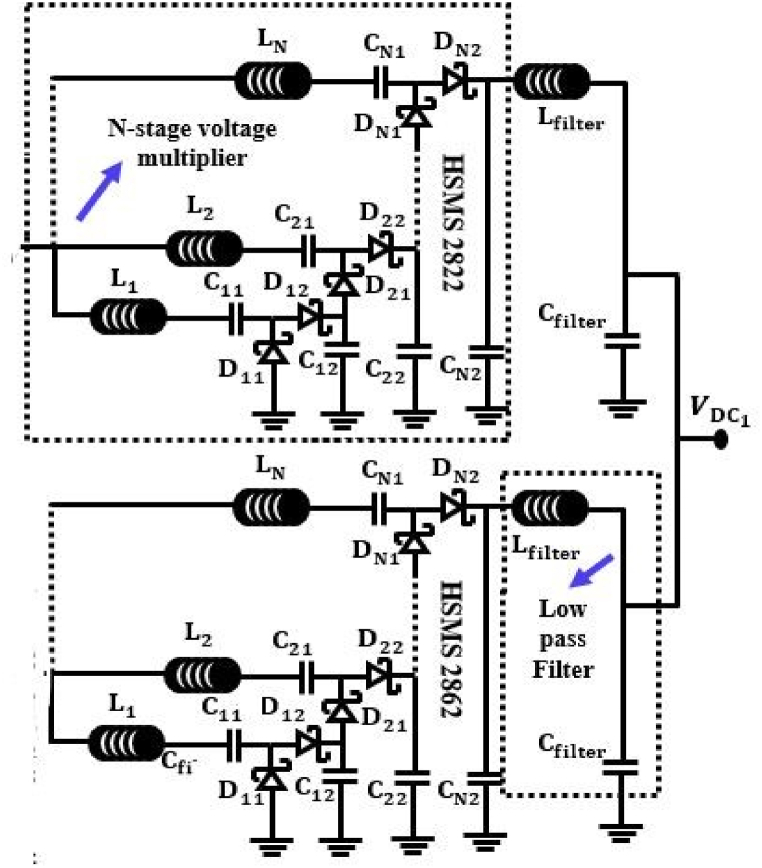
Schematic diagram of a N-stage voltage multiplier network.

The RF energy harvesting circuit works best with diodes that have the lowest turn-on voltage and switch very quickly. This is due to the high frequencies and low input RF power. For LPD and HPD, the Schottky diodes HSMS-2852 and HSMS-2822 from Avogo Technologies are chosen because for low input power, the HSMS-2852 performs better than the HSMS-2822. The efficiency of the conversion has gone up since an extra inductor was added. This inductor tries to store energy in a magnetic field during the negative cycle and release it during the next positive cycle. As a result, the current fluctuation may be limited by the opposite polarity of the voltage. This n-stage voltage multiplier raises the output voltage at the load while lowering the current. This might cause the energy storage capacitor to charge with unacceptable delays. Voltage gain decreases with increasing stages because of the parasitic effect of capacitors within each stage. A low-pass filter is utilized to eliminate harmonics of the alternating current frequency at the output. Fig. 10 depicts the sister concern of a voltage multiplier with a low-pass filter. Let's assume the input to the voltage multiplier is being fed a sinusoidal voltage, $V_{in}$, of frequency $f_{0}$ and amplitude $V_{0}$. For smooth output voltage, capacitors must have temporal constants much less than the input signal, that is, 1/(2π $R_{L}C$) << $f_{0}$, where C is the blocking capacitor and $R_{L}$ is the equivalent load resistance. An input-output feature is implied in this case by Ref. [81],

(7) $$\left(1+\frac{V_{\upsilon }}{R_{L}I_{S}}\right)\exp\left(\frac{V_{\upsilon }}{2NV_{T}}\right)=B_{0}\left(\frac{V_{0}}{V_{T}}\right)$$

where $V_{\upsilon }$, is the output voltage and $I_{S}$ is the voltage multiplier's output current. $V_{T}$ is the thermal voltage and $B_{0}$ is the modified Bessel function of zero order. Power dissipated by each diode, $P_{D}$ and power delivered to the load, $P_{L}$ can be used to determine the total input power, $P_{IN}$, essential to generate a certain amount of output voltage and power. Considering substrate losses, we can calculate the average input power as: [85],

(8) $$P_{IN}={2NI}_{S}V_{0}B_{1}\left(\frac{V_{0}}{B_{T}}\right)\exp\left(-\frac{V_{\upsilon }}{{2NV}_{T}}\right)+\frac{N}{2}{V_{0}}^{2}R_{sub}{\left(\omega_{0}C_{Sub}\right)}^{2}$$

where $R_{sub}$ and $C_{Sub}$ are the substrate resistance and capacitance, respectively.

The Q-factor of an LC matching network is proportional to the transformation ratio of the resistance and its expression is Q = $\sqrt{\frac{R_{eq}}{R_{A}}-1}$ [81]. In this expression, $R_{A}$ represents the antenna resistance and $R_{eq}$ represents the voltage multiplier's equivalent input resistance, considering the power consumption, that is, $R_{eq}$ = ${V_{0}}^{2}$/2 $P_{in}$ .

#### 4.4.1 Power management unit

**Fig. 11:**
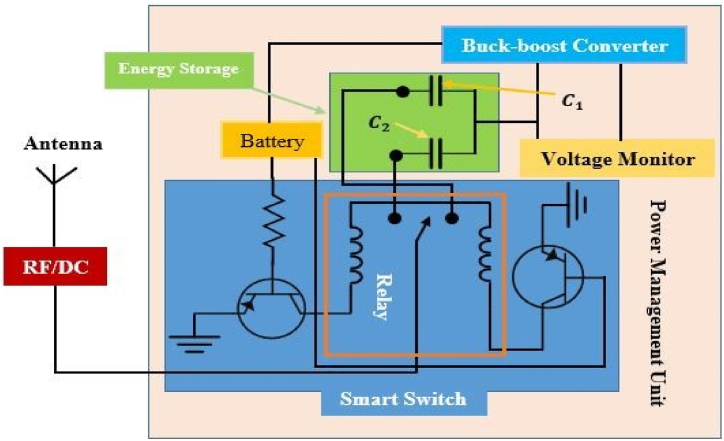
Smart switching system for storage unit.

The energy collected is then used by a power management unit (PMU) to supply the right amount of power to the load. Based on the design constraints, such as whether the load requires a controlled or accurate voltage or specific runtime, a customized power management system can be utilized. RF-EH systems prefer supercapacitors over batteries because of their high power density, rapid storage and handling, prolonged cycle life, compact size, and reduced cost. The major purposes of the power management unit (PMU) are to optimize the quantity of energy delivered from the antenna to the storage device in real time, store it in the storage unit, and finally supply the appropriate amount of power to the mobile phone battery. On a typical system, it is not guaranteed that the capacitor voltage will reach $V_{\max}$ in a single charging cycle. Fig. 11 depicts the smart switching system for the storage unit of the reported power management system.

The capacitors *C*_1_ (100 mF) and *C*_2_ (200 mF) are utilized as storage components in low and high energy settings, respectively. The capacitor *C*_2_ will be charged when the RF/DC conversion is performed by the HSMS 2822-based rectenna. Otherwise, the capacitor *C*_1_ will be charged. When the voltage of the capacitor, $V_{cap}$, reaches the maximum voltage, $V_{\max}$, of the capacitor, the booster network turns on and is still on until the voltage drops to $V_{\min}$. The energy storage device will deliver electricity to the mobile phone's battery if C (${V_{\max}}^{2}-{V_{\min}}^{2})/2>E_{Mobile}$ [86]. Additionally, it results in energy loss $E_{loss}=C{V_{residual}}^{2}/2$ which represents that the capacitor can harvest 67–100% of energy [87]. The energy level of the supercapacitor at time t is modeled as [87]:

(9) $$E_{Harvested}E_{Leak}E_{Load}$$

where $E_{Load}$ represents the amount of power supplied by the mobile phone battery and depends on the operation of charging mode e.g. in case no charging $E_{Load}$ = $E_{sleep}$. $E_{Leak}$ signifies the loss of energy due to the leakage current of the capacitor. $E_{Harvested}$ signifies the harvested energy by a rectenna network near an RF charging station. The harvested energy can model as [87]:

(10) $$E_{Harvested}=\int_{0}^{\tau }P_{RF}dt$$

where $P_{RF}$ denotes instantaneous RF received power level and *τ* represents the collocation time.

**Fig. 12:**
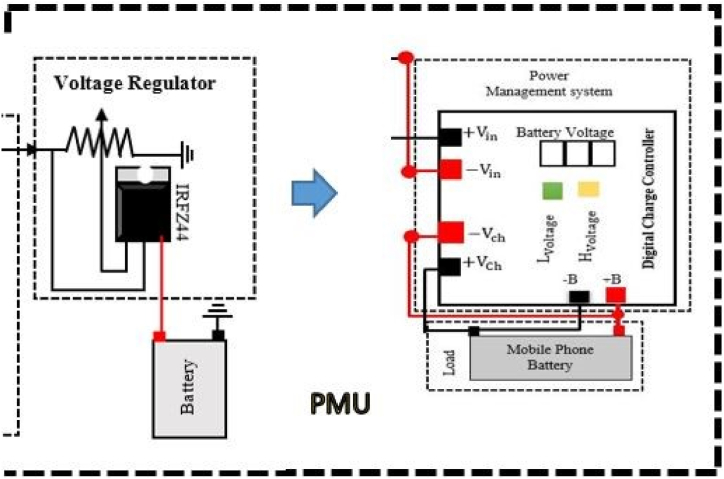
Schematic diagram of a power management unit.

In human applications, it is generally recognized that collocation time and node mobility exhibit statistically significant patterns. The average time a gadget spends near a charging station determines how much energy can be gathered. The proposed PMU is capable of persistently operating energy-intensive processes. The charger controller checks the current battery voltage, $V_{CurrentBattery}$ continuously and compares it with $L_{voltage}$. If the current voltage is less than $L_{voltage}$ the battery starts charging until the current voltage is equal to $H_{voltage}$. In the PMS, a regulated circuit is used to provide constant voltage as the battery requires. It increases the lifetime of the battery or helps protect the battery from damage. Fig. 12 depicts a power management system, including a digital charger controller.

## 5 Design of the proposed RF based wireless mobile phone charging system

### 5.1 Flow chart of the RF energy based wireless mobile phone charger

**Fig. 13:**
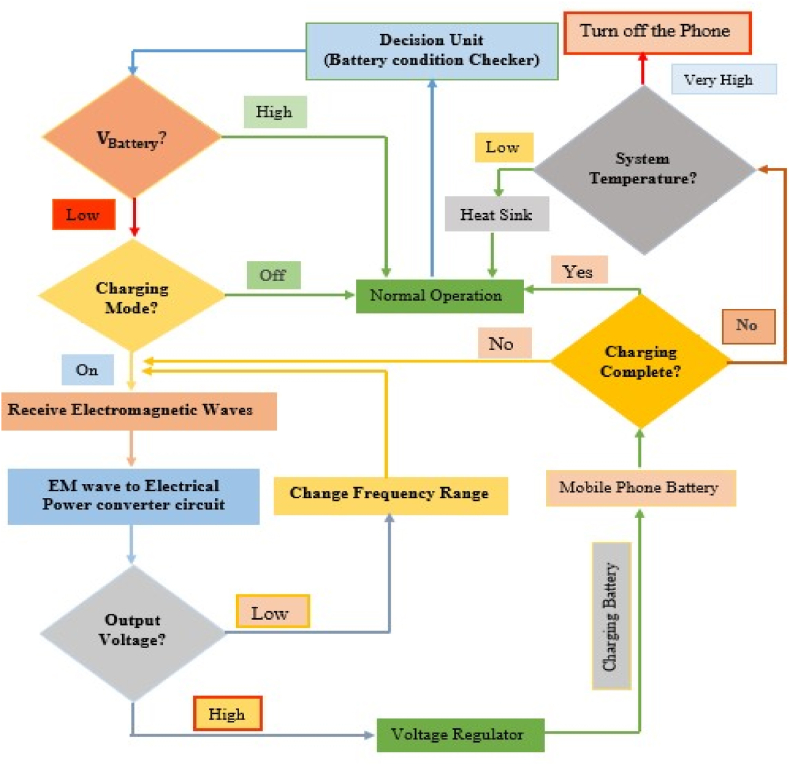
Flow chart of the proposed charging system.

A diagrammatic representation of the suggested model is shown in Fig. 13, which gives an overview of the model. The decision unit continuously sends information about the battery condition. If the current voltage of the battery is low, the antenna will act as a charger as well as a transmitter or receiver. Otherwise, the antenna will act as a receiver and/or transmitter. Depending on the matching circuit, the antenna will normally receive high-frequency electromagnetic waves. These waves will pass through the proposed RF/DC power converter circuit. Through the use of a rectenna circuit, the RF signal is transformed into DC power. Rectenna circuits are composed of a rectifier circuit, a voltage multiplier, and a low-pass filter. A regulator circuit is used to give the cell phone battery the right amount of power at the right time. The antenna will take in as much low-frequency or multi-band (like 0.9 GHz, 1.8 GHz, 2.1 GHz, and 2.45 GHz) radiation from the radiating components around it as it needs to make enough RF/DC voltage to charge the battery. The antenna will be in charging mode until the battery is completely charged. During charging, if the temperature of the system is in a sustainable range or is low, the dissipated energy as heat from the system will be received by the heat sink, and the charging mode will be active. Otherwise, the mobile phone will be off. The ultimate outcomes of the simulated results were discussed in Section VI.

### 5.2 Algorithm of proposed rectifier (impedance matching) circuit

1. count = 0;
2. Check the antenna operating mode.
3. If charging mode is on, then go to level 1.
4. else normal operation will continue.
5. level 1:
  i. Compare $V_{rectifier}$ with ${V_{in}}_{\min}$.
  ii. if ${V_{in}}_{\min}$ ${<V}_{rectifier}$, then go to level 2.
  iii. else multiply $V_{rectifier}$.
6. level 2: change preferred network:
  i. for i = 1 to n:
  ii. short *L*_*i*_ and go to level 1.
  ii. count ++
  iii. end for.
7. if [(count = = n) && ${V_{in}}_{\min}$ *<* $V_{rectifier}$], then go to level 3.
8. else go to level 1.
9. level 3: [Emergency charging/only charging mode]
  iv. for i = 1 to n:
  v. short $L_{i}$ and $\sum_{i=1}^{n}V_{rectifier}$
  vi. end for.
  vii. compare $\sum_{i=1}^{n}V_{rectifier}$ with ${V_{in}}_{\min}$.
  v. if $\sum_{i=1}^{n}V_{rectifier}=={V_{in}}_{\min}$ , then go to level 1.
  v. else change your location and go to level 1.

Here, $V_{rectifier}$, ${V_{in}}_{\min}$ indicate rectified voltage, required minimum voltage of the voltage multiplier to amplify. count indicates the number operating networks (2G, 3G, 4G, 5G etc.)

### 5.3 RF energy based wireless charging system design and analysis

Most of the time, you need an array of many antennas because one antenna isn't good enough for communication or isn't enough to gather enough RF power, which is then turned into RF/DC power and sent to the device so it can work. There are two ways to put rectennas together in an array, and each has its pros and cons. In one configuration, each antenna in an array is compensated by an antenna with a unique rectifier to harvest DC power. These antennas are combined in a series and/or parallel configuration. In another configuration, for a single rectifier, multiple antennas can be arranged. A mobile phone's existing antenna can be single-band (multiple antenna) or multiband. In this study, we discussed both types of antenna for RFEH.

#### 5.3.1 Single band multiple antenna based RFEHS

**Fig. 14:**
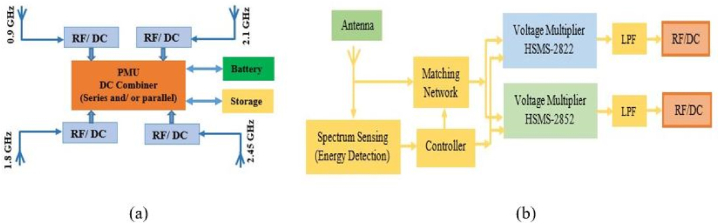
Block diagram of the proposed RFEH system from multiple single band antenna.

For the efficient implementation of the intelligent energy harvesting circuit, as illustrated in Fig. 14, a rectenna system was investigated using modeling and simulated data. The basic idea behind the system was to combine multiple antennas so that radio frequency energy from different frequencies could be collected. To fix the problems with frequency hopping and signal frequency tuning, the circuits need to be changed with the help of a controller. You could also use a wideband receiver antenna, which can pick up signals from many different places. Schematic configurations of the power management system and the RF/DC converter are shown in Fig. 14(a) and (b), respectively. All antennas were equipped with an RF power detector IC (LTC5505). The output power and RF power detector were used to automatically tune the matching circuit. An empirical method known as “annealing” was used to fit the antenna. In the voltage multiplier section, an N-stage (4 to 7-stage) voltage multiplier composed of Schottky diodes (HSMS-2822 and HSMS-2852) is described. The power management circuit, also known as the charging controller, serves as both a switch and a reservoir. If the battery is low, it charges the battery, and if only the device's terminal operations are needed in battery-free devices, it operates only those functions.

##### 5.3.1.1 Rectification section of the RFEHS based on multiple antenna

**Fig. 15:**
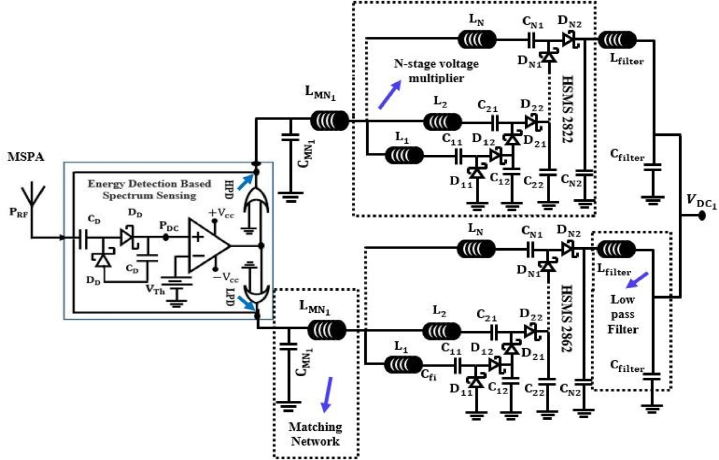
Schematic circuit diagram of the proposed RFEH system from a single band antenna.

Schottky diodes, which work with zero bias, are good for rectifier circuits at high frequency ranges and low RF power densities, even though they receive very little RF power and work at high frequency ranges. Schottky diodes are frequently employed to increase PCE from a half wave due to their low substrate losses and high switching frequency. The way a diode's input and output work in a rectifier circuit is the basis for converting an RF signal to a DC signal. To multiply the voltage after it has been fixed, an N-stage voltage multiplier with an inductor is used. It serves to store energy in a magnetic field. By discharging, the couple moves the energy that was built up during the negative half to the positive half (L, C). Moreover, a passive low pass filter is set up in the circuit design to eliminate ripple and give smooth DC power. In this set-up, each antenna in an array is a separate antenna with a rectifier that can gather DC power on its own. The schematic circuit diagram of the proposed RFEH system from a single band antenna is depicted in Fig. 15.

##### 5.3.1.2 Power mangement unit of RFEHS based on multiple antenna

**Fig. 16:**
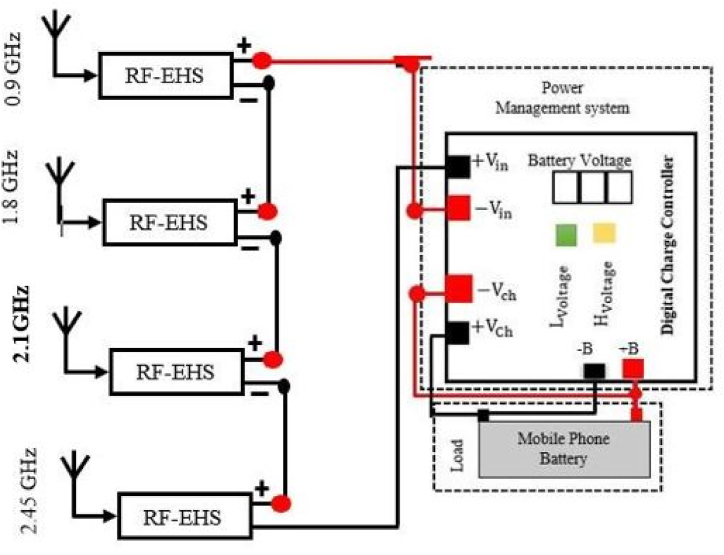
Schematic diagram of the proposed power management system with multiple single band antenna.

The energy collected is then used by a power management unit (PMU) to send the right amount of power to the load. Based on the design constraints, such as whether the load requires a controlled or accurate voltage or precise running times, a customized power management system can be utilized. RF-EH systems prefer supercapacitors due to their high power density, quick storage and handling, extended cycle life, compact size, and lower cost compared to batteries. The major purposes of the power management unit (PMU) are to optimize the quantity of energy delivered from the antenna to the storage device in real time, store it in the storage unit, and finally supply the appropriate amount of power to the mobile phone battery. The charger controller checks the current battery voltage continuously and compares it with $L_{voltage}$. If the current voltage is less than $L_{voltage}$ the battery starts charging until the current voltage is equal to $H_{voltage}$. In the PMS, a regulated circuit is used to provide constant voltage as the battery requires. It increases the lifetime of the battery or helps protect the battery from damage. Fig. 16 depicts a schematic of the proposed power management system using multiple single-band antennas. In this section, we design a compact rectenna array with four separate rectennas using the configuration where each rectenna has its own antenna and rectifier. The rectennas are combined in series configuration.

#### 5.3.2 Multiband antenna based RFEHS

**Fig. 17:**
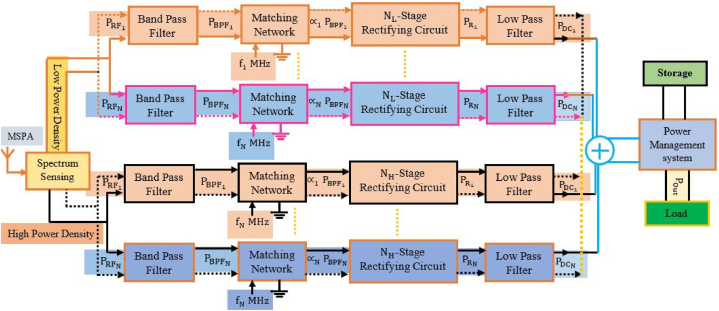
Block diagram of the proposed RFEHS based on multiband antenna.

This study demonstrates a novel microstrip antenna that is based on a stepped impedance resonator (SIR). The antenna that is given is chosen so that it will work with future wireless communication systems that use more than one standard. A block diagram of the proposed RFEHS base on multiband antenna is shown in Fig. 17.

##### 5.3.2.1 Rectification section of the RFEHS based on multiband antenna

**Fig. 18:**
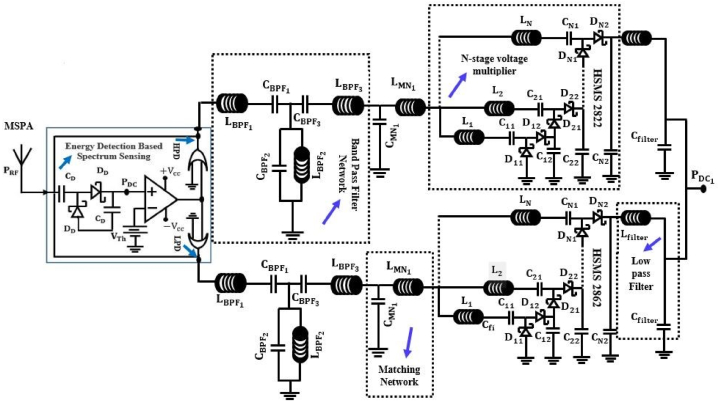
Schematic circuit diagram of the proposed RFEHS based on multiband antenna.

The operating functions of this section are identical to those of an RFEHS rectification section with a multiband antenna. A band pass filter (Pi-matching) is designed in the circuit design to operate the antenna at a specific frequency range and is connected between each rectifier circuit and the antenna. This makes the circuit complexity four times greater than the conventional rectification circuit. Fig. 18 depicts the schematic circuit diagram of a rectenna based on multi-band antenna.

##### 5.3.2.2 PMU of the RFEHS based on multiband antenna

**Fig. 19:**
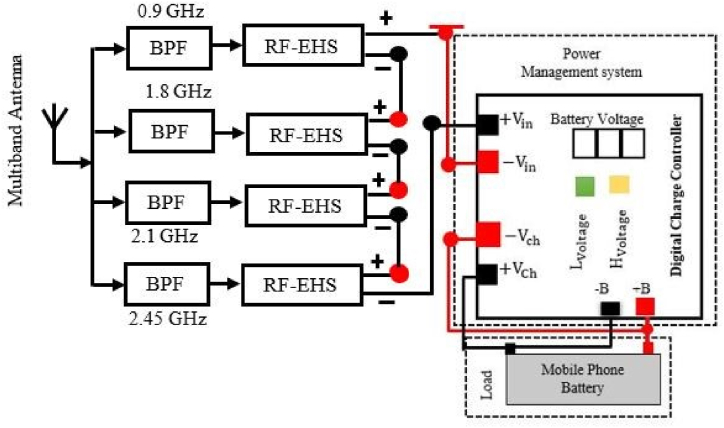
Block diagram of the proposed RFEHS based on multiband antenna.

The working principle of a power management unit (PMU) based on a multiband rectenna is similar to that of a PMU based on a single-band rectenna. Fig. 19 shows a schematic diagram of a rectenna system that uses a multi-band antenna including PMU. In this section, we also design a compact rectenna array with four separate rectennas using the configuration where each rectenna has its own antenna and rectifier. The rectennas are combined in series configuration.

#### 5.3.3 Antenna design and development

The primary function of a microstrip antenna is to radiate. Its size must thus be around g/2, where g is the guided wavelength.

##### 5.3.3.1 Single band microstrip patch antenna

**Fig. 20:**
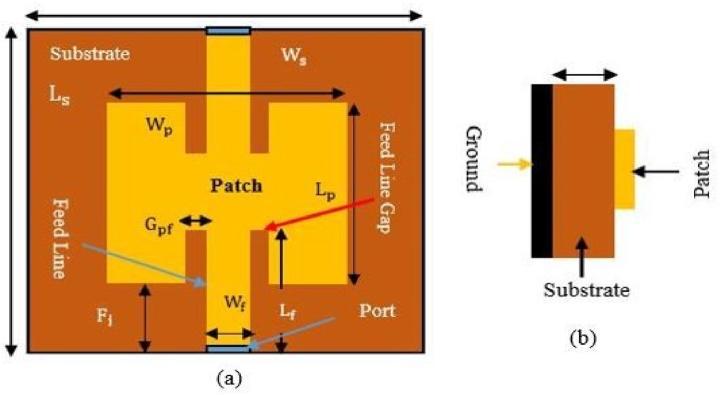
Geometry of the proposed rectangular microstrip patch antenna.

**Fig. 21:**
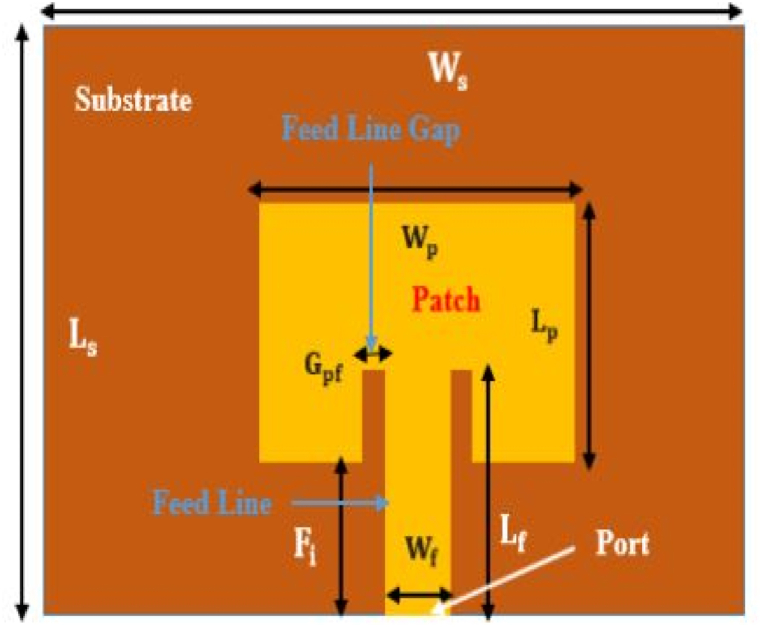
Rectangular microstrip patch antenna.

The suggested antennas are printed on a substrate made of FR-4 dielectric material, with a thickness of 1.6 mm and a permittivity of 4.4. The frequencies of operation for each of the single-band antennas are as follows:

0.9 GHz, 1.8 GHz, 2.1 GHz, and 2.45 GHz. For example, the first radiating patch antenna is 101.4 mm long and 79.22 mm wide, while the second patch, which connects to the top of patch one, is 39.41 mm long and 50.68 mm wide. This portion is in charge of the operations that take place at 0.9 and 1.8 GHz. Patch 4th is related to the third radiating element and has dimensions of 28.81 mm in length and 37.23 mm in width. The length of the third radiating antenna is 33.7 mm, and its breadth is 43.44 mm. This portion emits frequencies between 2.1 and 2.45 GHz. The designed antennas are differentially fed, and the matching impedance of 50 Ω can be seen clearly. Fig. 20 illustrates the geometry of the reported single band antenna. HFSS was used in the process of designing and simulating this proposed multiband antenna (see Fig. 21).

##### 5.3.3.2 Reconfigurable multi-band microstrip patch antenna

**Fig. 22:**
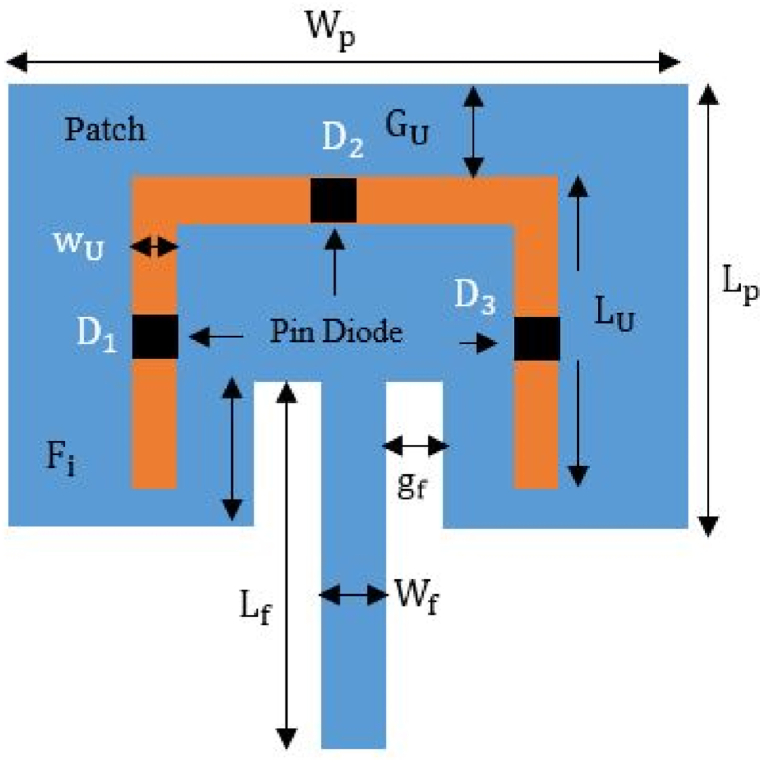
Reconfigurable U-shaped microstrip patch antenna.

This paper demonstrates a novel reconfigurable multi-band microstrip antenna. The proposed flexible and stretchable antenna is 34.78 mm × 58.84 mm in size and the length of the U-shape is 30 mm. For non-stretchable antenna the size of the patch is 50.68 mm × 101.4 mm and the length of the U-shape is 40 mm. In the proposed design, the ground plane is modified to support ISM applications using a rectangular patch and a U-shaped slot for a multiband antenna. In the middle of each slot, three-pin diodes (D1, D2, and D3) are inserted. By turning the diodes on and off, this antenna can be operated at a certain frequency band for wide range of purposes. Modifying one substrate layer and a common ground plane results in the desired resonance frequency. The proposed layout reduces overall dimensions by making adjustments to the ground plane. The effectiveness of an antenna is enhanced by modifying the ground. Fig. 22 depicts the geometry of the designed reconfigurable microstrip patch antenna. A single-fed line approach that exhibits good matching at 50 Ω is used to feed the rectangular radiating patches. The impedance matching, peak gains, and reflection coefficient simulation results all display satisfactory stability. FR-4 is the substrate used to fabricate the antenna, and the simulation tools used are HFSS, ADS, ABAQUS and MATLAB.

##### 5.3.3.3 Design parameters of the proposed single band antenna

**Fig. 23:**
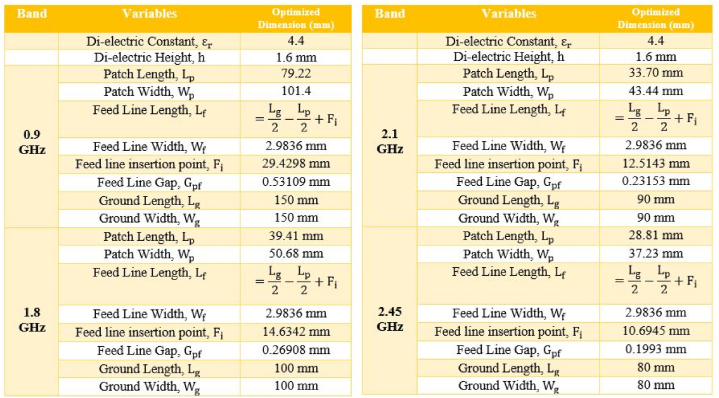
Dimension of single band multiple antenna (solid patch and solid ground).

Fig. 23 depicts the dimensions of single band multiple antennas, which consist of a dielectric substrate, a patch, and a microstrip feed line. The above-described dimensions of the FR-4 Dielectric substrate are used to isolate the rectangular patch from the ground plane.

**Fig. 24:**
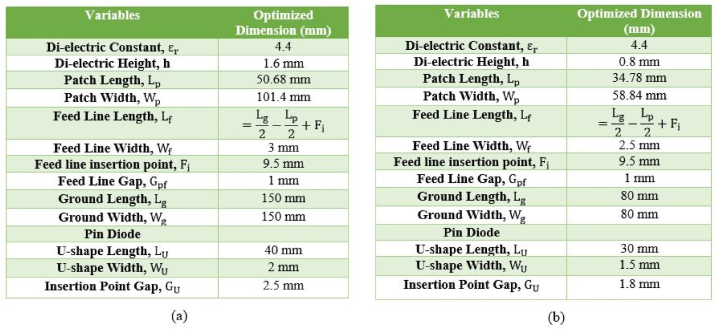
Dimension of reconfiguriable multiband antenna (a) non stretchable antenna (solid patch and solid ground) (b) stretchable antenna (meshed patch and meshed ground).

Dimensions of the suggested non-stretchable and stretchable multiband antennas are shown in Fig. 24(a) and 24(b) respectively. The above described dimensions of the FR-4 dielectric substrate are used to isolate the rectangular patch from the ground plane. Altering the values of the substrate, patch, and ground dimensions can enable the best possible outcomes. Moreover, altering the structure of the substrate and patch can enable the best possible outcome: a stretchable and flexible antenna.

#### 5.3.4 Analysis and design equations

**Fig. 25:**
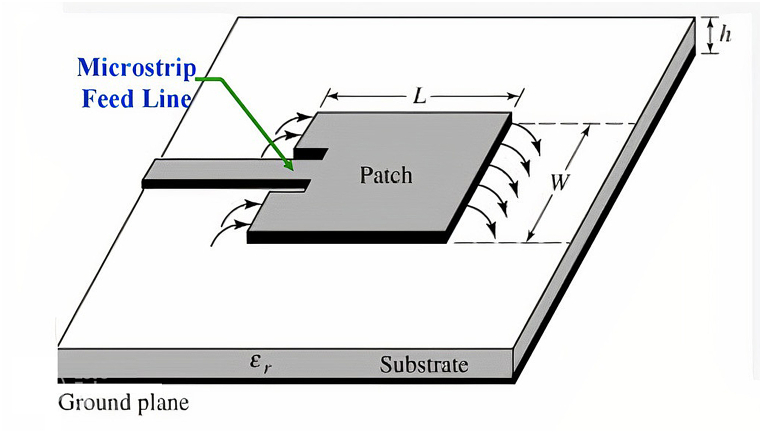
Rectangular microstrip patch antenna line feed [89].

Fig. 25 shows the schematic design of a rectangular microstrip patch antenna. We apply equations from Equation (11), (12), (13), (14), (15), (16), (17), (18), (19), (20), (21) [88] to construct a rectangular microstrip patch antenna with line feeding. The first and foremost thing is operating frequency or resonant frequency, $f_{r}$ or, $f_{0}$. Next, take the value of substrate height, h and the dielectric constant, $\varepsilon_{r}$.

Step 1: Calculation of the patch width (w):

(11) $$w=\frac{c}{{2f}_{r}}\sqrt{\frac{2}{\varepsilon_{r}+1}}$$

Step 2: Determine the effective value of the dielectric constant. This occurs as a result of the height as well as the dielectric constant of the substrate, in addition to the calculated width of the patch antenna.

(12) $$\varepsilon_{eff}=\frac{\varepsilon_{r}+1}{2}+\frac{\varepsilon_{r}+1}{2}{\left[1+12\frac{h}{w}\right]}^{-\frac{1}{2}}$$

Step-3: Calculation of the length that will be effective, $L_{eff}$:

(13) $$L_{eff}=\frac{c}{2f_{c}\sqrt{\varepsilon_{eff}}}$$

Step-4: The determination of the length extension's calculation, Δ L:

(14) $$\frac{\Delta L}{h}=0.412\frac{\left(\varepsilon_{r}+0.3\right)\left(\frac{w}{h}+0.264\right)}{\left(\varepsilon_{eff}+0.3\right)\left(\frac{w}{h}+0.8\right)}$$

Step-5: Calculation of the effective length:

(15) $$L_{eff}=\frac{C}{2f_{c}\sqrt{\varepsilon_{r}}}-2\Delta L$$

Step-6: Calculation of actual length of the patch:

(16) $$L_{eff}-$$

Step-7: Height of the patch calculation:

(17) $$h_{patch}=\frac{0.3C}{2\pi f_{c}\sqrt{\varepsilon_{r}}}$$

Step-8: Width of the feedline, $w_{feedline}$:

(18) $$w_{feedline}\approx 2.932\;mm$$

where $Z_{0}=50\Omega \text{.}$

Step-9: Calculation of ground length:

(19) $$L_{ground}\approx 2L$$

Step-10: Calculation of ground width:

(20) $$w_{ground}\approx 2w$$

Step-11: Length of the feedline, $L_{feedLine}\text{:}$

(21) $$L_{feedLine}=\frac{6h}{2}$$

here, *f*_0_ represents the Resonance Frequency, w represents the Width of the Patch, L indicates the Length of the Patch, and h is the thickness of the Patch, $\varepsilon_{r}$ is the relative permittivity of the dielectric substrate, while c stands for the speed of light: 3 × 10^8^. Gap between patch and inset feed, $G_{pf}$ ≈1 mm.

#### 5.3.5 Fabrication method

**Fig. 26:**
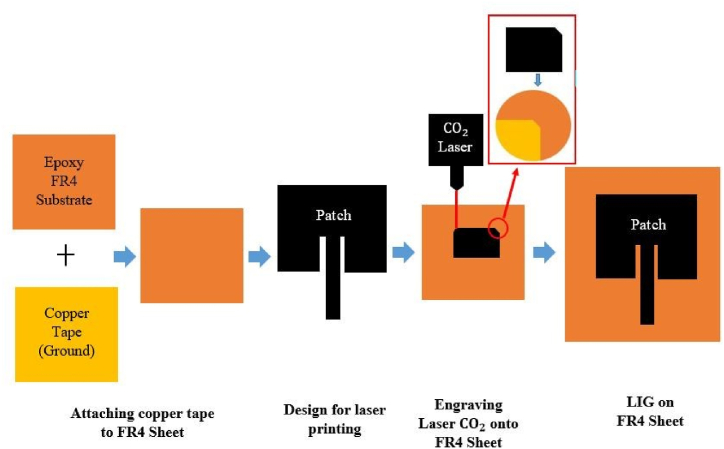
Step-by-step illustration of the fabrication process of the LIG-based MPA (solid metal-plated).

In this study, we build a flexible, miniaturized strain sensor antenna that is sensitive to both compressive and tensile strain and is fabricated using LIG printing. Under a specific laser threshold power, the epoxy FR4 sheet behaves as an insulator with a substantially larger sheet resistance. The sheet resistance of etched porous graphene is reduced and its conductivity is enhanced as the applied power is increased. The substrate sheet works as an insulator with considerably increased electrical resistance under a specific laser power threshold. As the power is gradually increased, the sheet resistance of etched porous graphene decreases and the conductivity increases. Using a laser to illuminate the FR4 sheet generates a highly flexible, porous material suitable for strain sensing. Fig. 26 depicts the process of fabricating the LIG antenna-based sensor with a *CO*_2_ laser of optimal power of 3 W. Before manufacturing the LIG, the FR4 sheet has adhesive copper tape on only one side. On the FR4 sheet, the *CO*_2_ laser with the requisite power and speed induces graphene via engraving. Finally, the *CO*_2_ laser immediately cuts a contour based on the parameters required for the antenna. The most significant microfabrication challenges can be circumvented by preserving dimension uniformity, hence reducing the time required for mass fabrication.

**Fig. 27:**
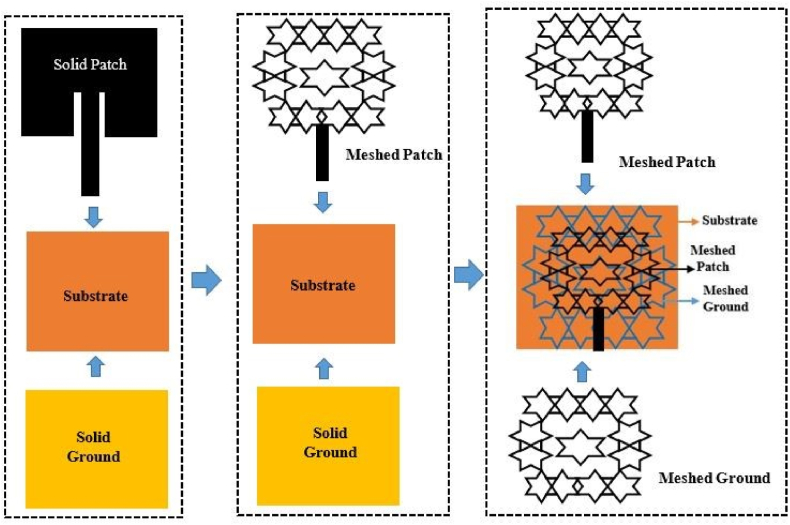
Step-by-step illustration of the fabrication process of enhancing stretchability by converting the solid patch to a mesh structure.

Fig. 27 depicts the schematic perspective of enhancing the stretchability of microstrip antennas by transforming the solid patch to a mesh structure. First, the concept of a LIG-based MPA is shown in Fig. 26. Based on this approach, the surface of the patch was printed with a mesh pattern to enhance its stretchability. Subsequently, the ground plane was also redesigned using a mesh configuration, which made it possible to stretch it up to 100%. The finite element analysis was performed using the software ABAQUS (ABAQUS 6.14, ABAQUS Inc.) to determine the deformed configuration and strain distribution of the reported antenna applied to a specified tensile strain.

## 6 Simulated results and discussion

### 6.1 Antenna performance

**Fig. 28:**
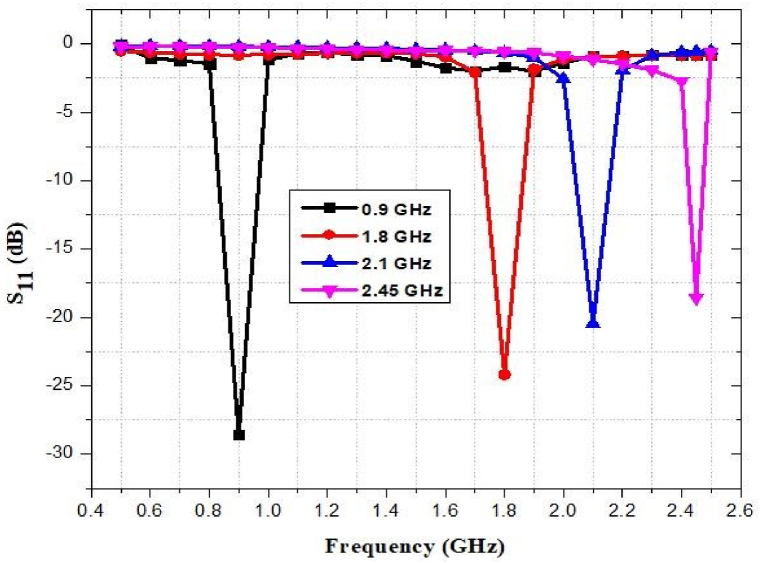
Return loss of the proposed antenna.

The simulated return loss of the reported antenna is illustrated in Fig. 28. Within the frequency operation range of 0.9–2.45 GHz, the antenna is selected to complement the system well enough so that less than one percent of the broadcast signal is evidenced back to the antenna (i.e., below 20 dB). The antenna exhibits a return loss of −28 dB at 0.9 GHz, −24 dB at 1.8 GHz, −20 dB at 2.1 GHz, and −17 dB at 2.45 GHz. The lowest return loss is recorded at 2.45 GHz (−17 dB). Return Loss is less than −10 dB across all frequency ranges, which is not ideal for an antenna, but still meets the design criteria.

### 6.2 VSWR of the proposed antenna

**Fig. 29:**
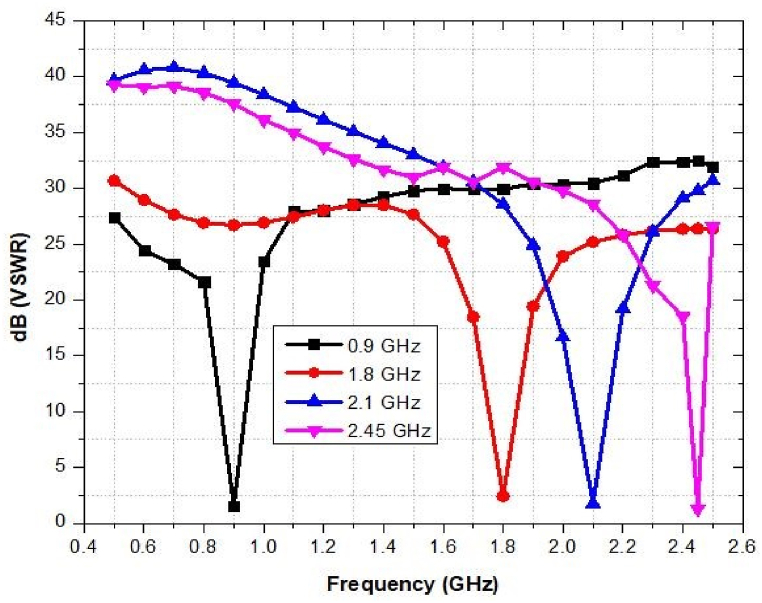
VSWR of the proposed antenna.

The simulated VSWR of the designed antenna is depicted in Fig. 29. The antenna's VSWR at 0.9 GHz, 1.8 GHz, 2.1 GHz, and 2.45 GHz is 1.25 dB, 1.58 dB, 1.38 dB and 1.38 dB, respectively. Even though VSWR is less than 2 for all frequency ranges, it still meets the design requirements for an antenna.

### 6.3 3D-radiation pattern of the prospective antenna

**Fig. 30:**
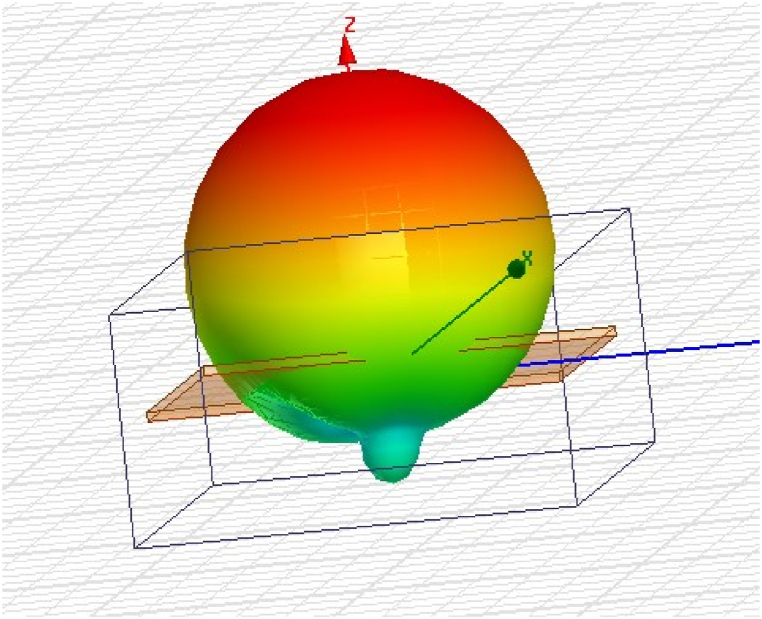
3D-radiation pattern of the proposed multiband antenna.

The 3D-Radiation pattern of the reported antenna clearly demonstrates its great coverage and the 360° reach of its radiations. Fig. 30 shows the radiation parameters of the proposed antenna as energy distributions in space. Depending on the frequency, the form or pattern of the radiation alters. At frequencies of 0.9 GHz, 1.8 GHz, 2.1 GHz, and 2.45 GHz, the radiation pattern of the proposed antenna is almost omnidirectional. So, the antenna design is very good for a RF-EHS because it meets the criteria needed to get the most RF energy.

### 6.4 2D-radiation pattern of the proposed antenna

**Fig. 31:**
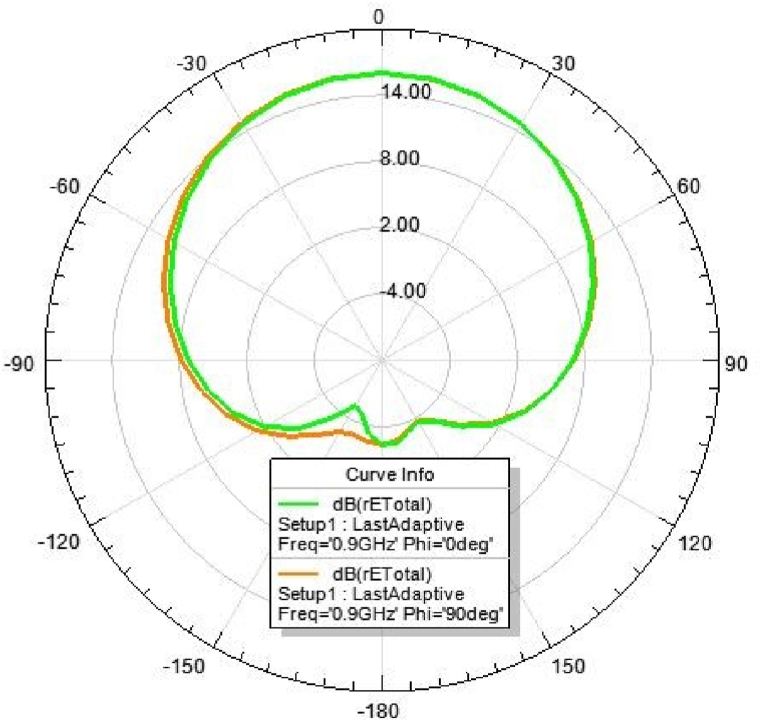
2D-Radiation pattern of at 0.9 GHz (in E-plane and H-plane).

**Fig. 32:**
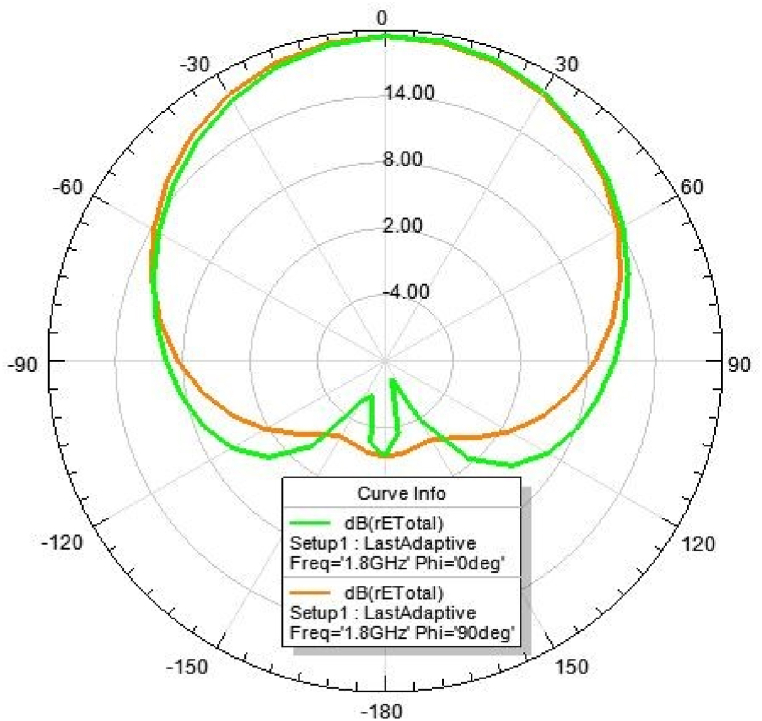
2D-Radiation pattern at 1.8 GHz (in E-plane and H-plane).

**Fig. 33:**
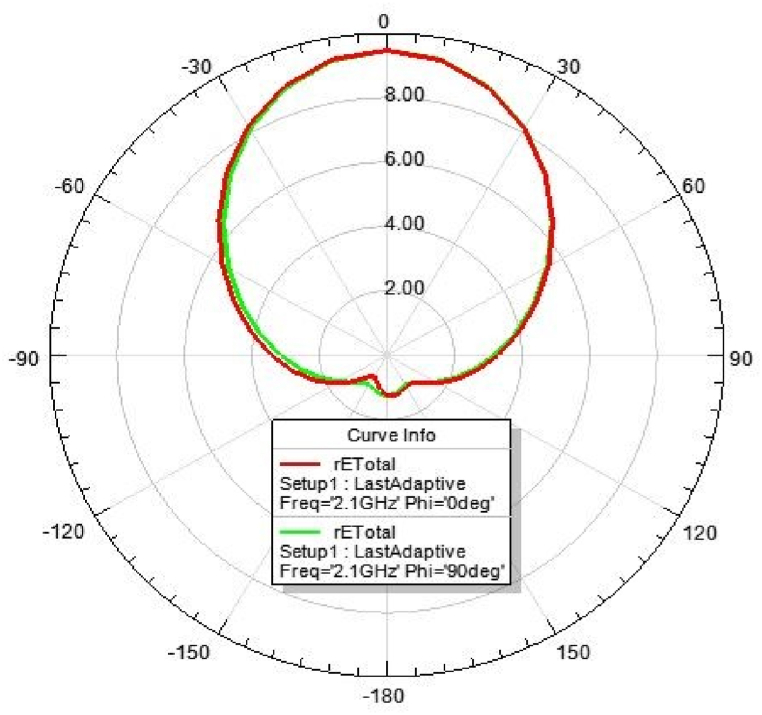
2D-Radiation pattern at 2.1 GHz (in E-plane and H-plane).

**Fig. 34:**
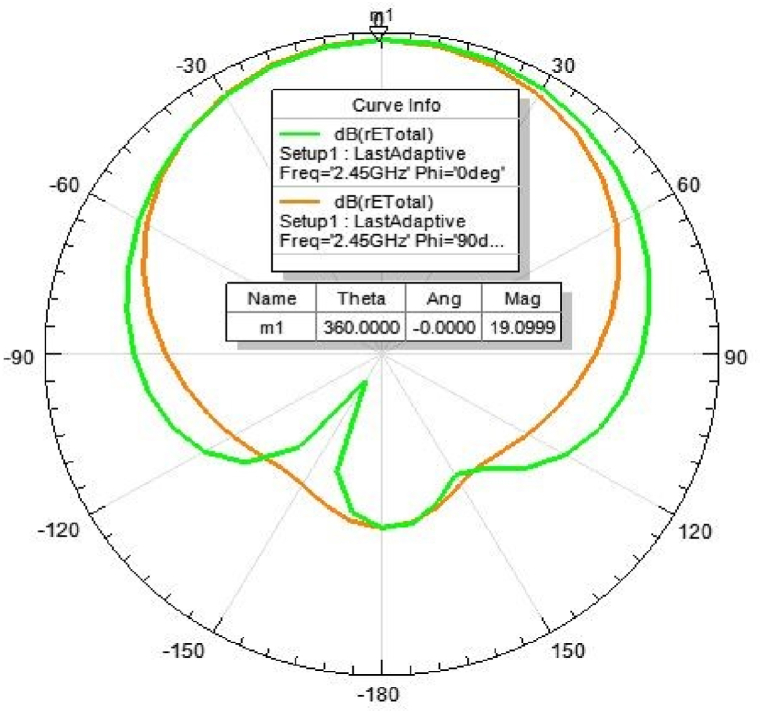
2D-Radiation pattern at 2.45 GHz (in E-plane and H-plane).

Fig. 31, Fig. 32, Fig. 33, Fig. 34 depicts the E-plane and H-plane radiation patterns of the reported antenna at 0.9 GHz, 1.8 GHz, 2.1 GHz, and 2.45 GHz. respectively. The designed multi-band antenna has a nearly circular distribution of polarization. The antenna exhibits stable radiation coverage in both the E and H planes, according to the simulation results.

*2D Radiation pattern at 0.*9 GHz: The result of the simulation of the 2D radiation pattern is depicted in Fig. 31.

*2D Radiation pattern at 1.*8 GHz: The result of the simulation of the 2D radiation pattern is depicted in Fig. 32.

*2D Radiation pattern at 2.*1 GHz*:* The simulated result of the 2D-radiation pattern is depicted in Fig. 33.

*2D Radiation pattern at 2.*45 GHz*:* The result of the simulation of the 2D radiation pattern is shown in Fig. 34.

### 6.5 Gain of the proposed antenna

**Fig. 35:**
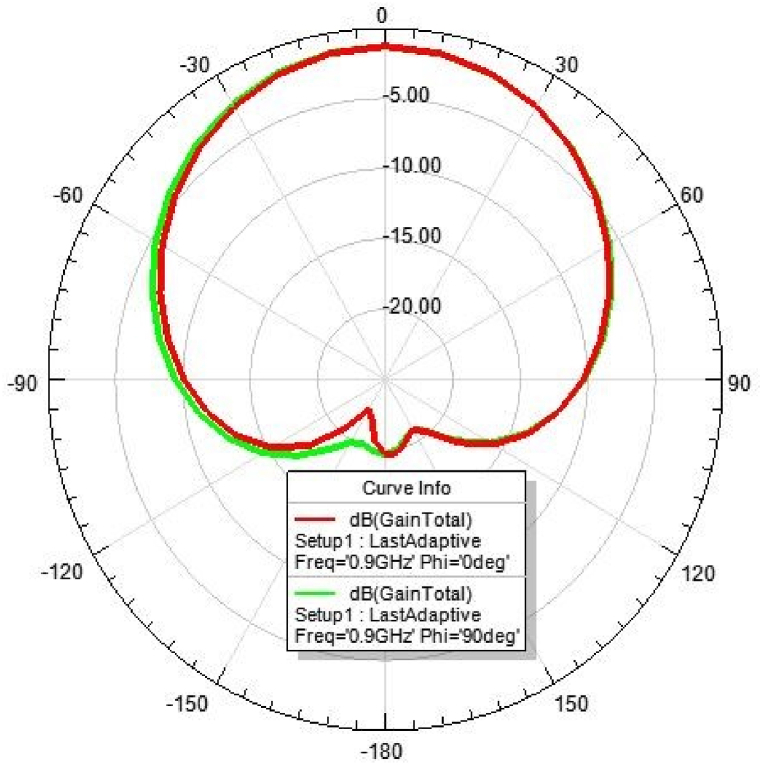
Gain at 0.9 GHz.

**Fig. 36:**
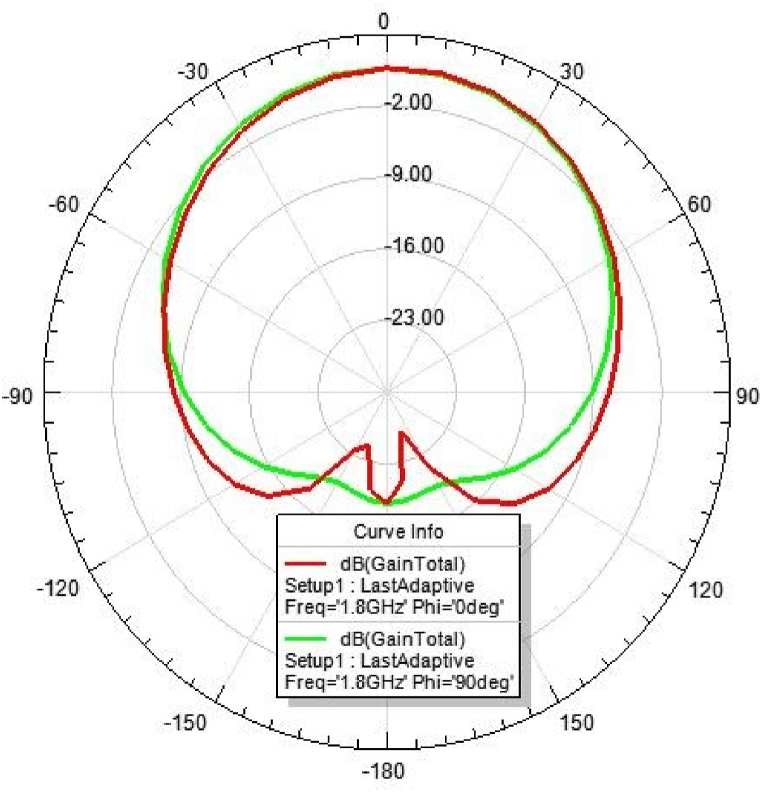
Gain at 1.8 GHz.

**Fig. 37:**
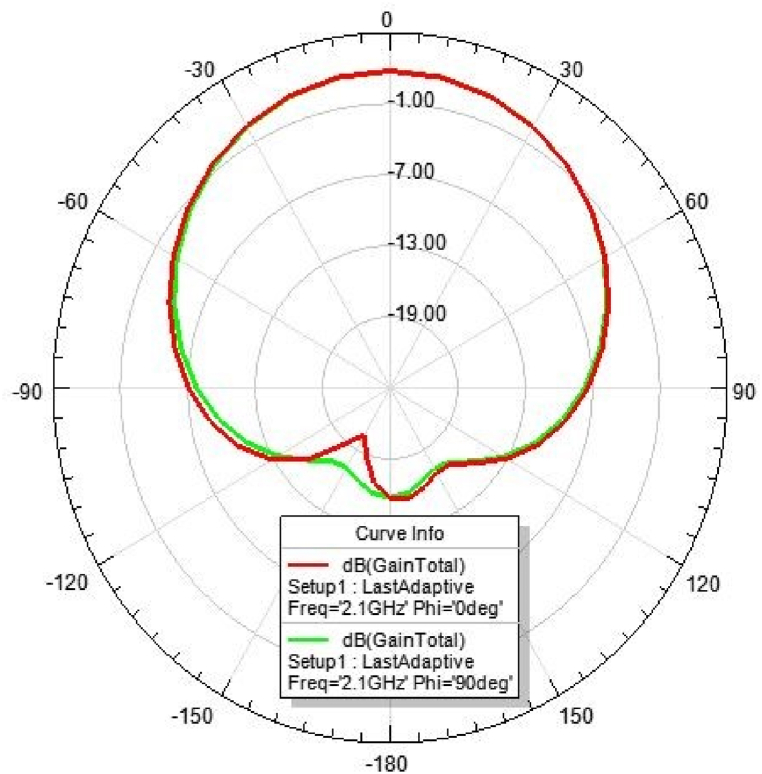
Gain at 2.1 GHz.

**Fig. 38:**
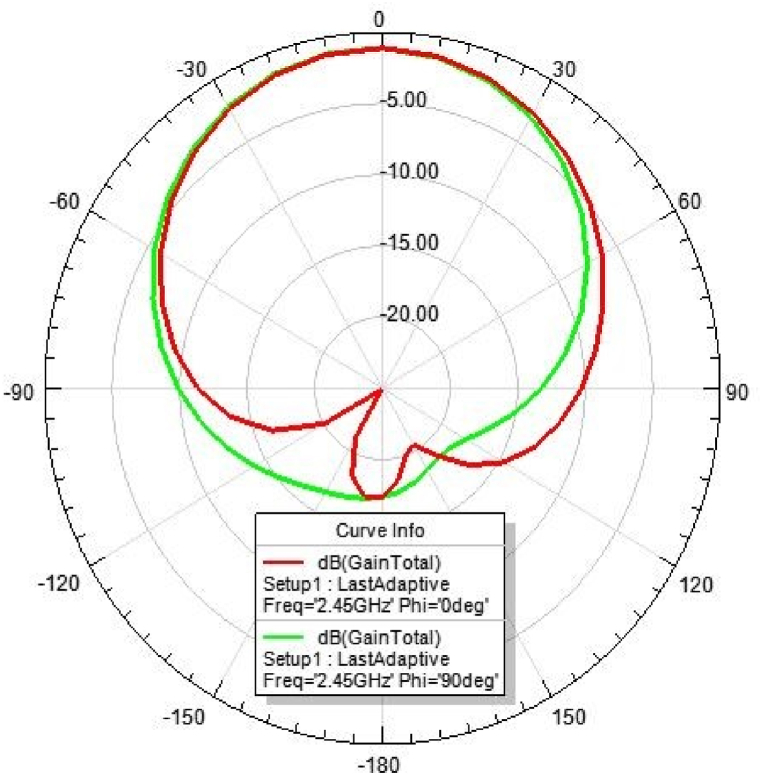
Gain at 2.45 GHz.

Fig. 35, Fig. 36, Fig. 37, Fig. 38 depict the individual gain of the proposed antenna.

### 6.6 Directivity of the proposed antenna

**Fig. 39:**
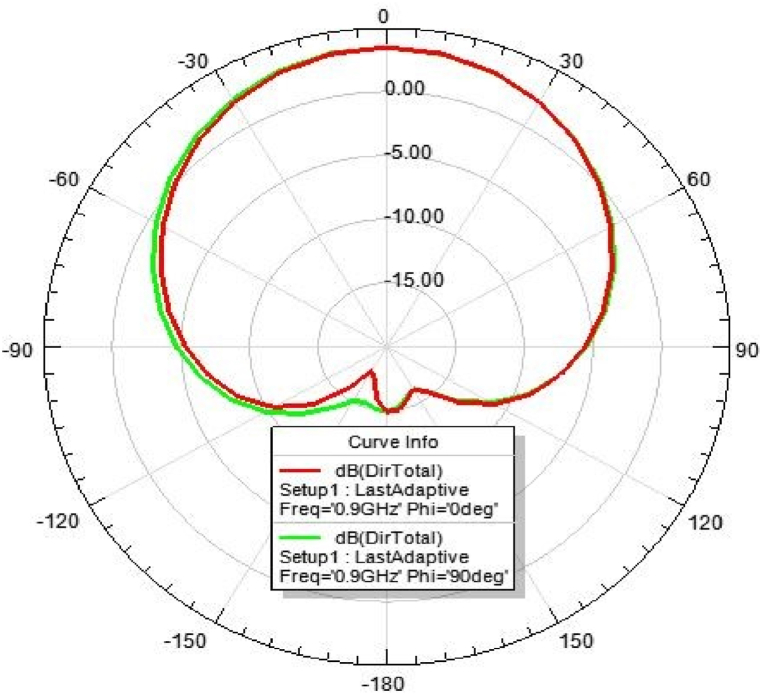
Directivity at 0.9 GHz.

**Fig. 40:**
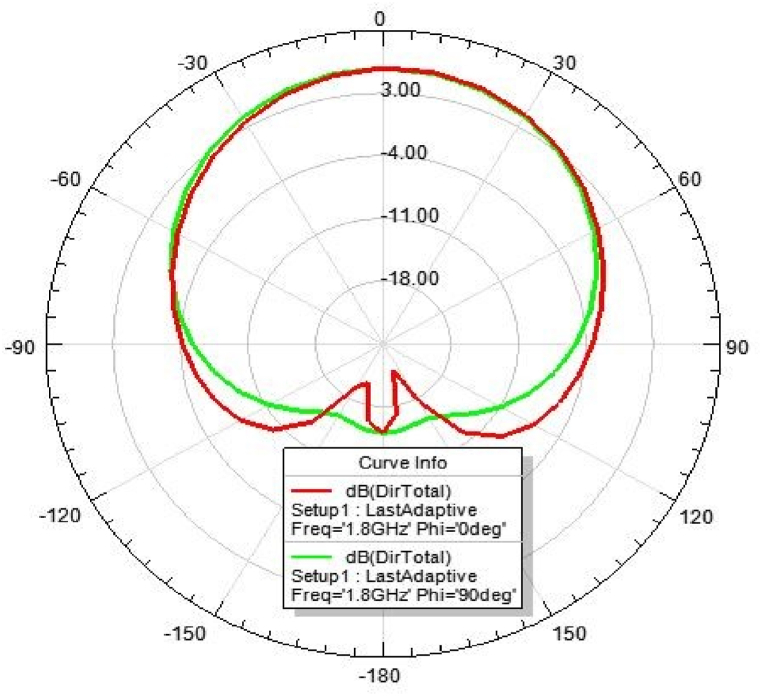
Directivity at 1.8 GHz.

**Fig. 41:**
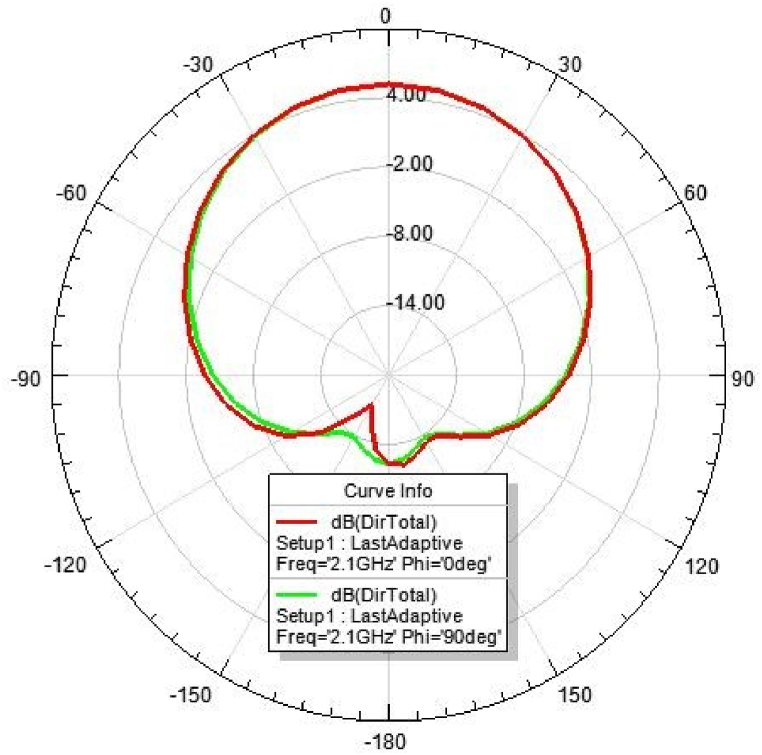
Directivity at 2.1 GHz.

**Fig. 42:**
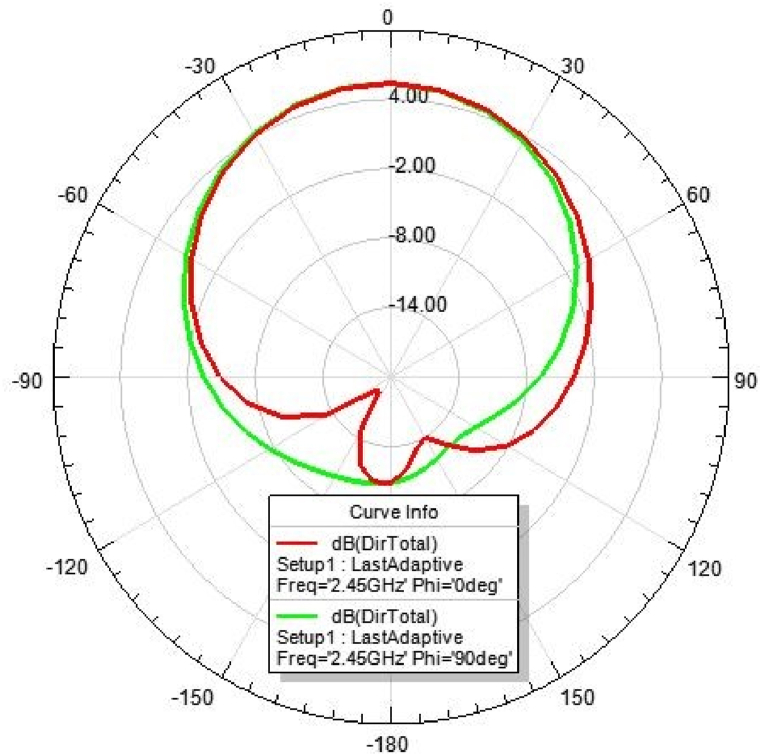
Directivity at 2.45 GHz.

Fig. 39, Fig. 40, Fig. 41, Fig. 42 depicts the individual gain of the proposed antenna.

### 6.7 Gain against frequency of the proposed antenna

**Fig. 43:**
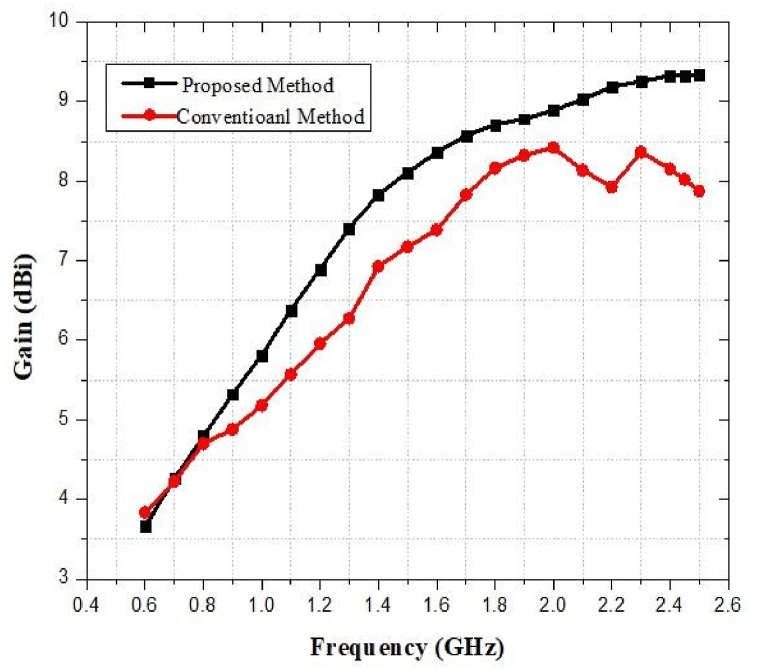
Gain against frequency of the proposed multiband antenna.

The gain that was estimated for the suggested rectangular microstrip patch antenna is depicted in Fig. 43. The antenna gain is 3.7 dBi at the 0.9 GHz, 8.7 dBi at the 1.8 GHz, and 9.2 dBi at the 2.1 GHz and 9.3 dBi at 2.45 GHz. When the source location is known, an antenna with a high voltage gain is needed so that the most wireless power can be received.

### 6.8 Radiation efficiency against frequency

**Fig. 44:**
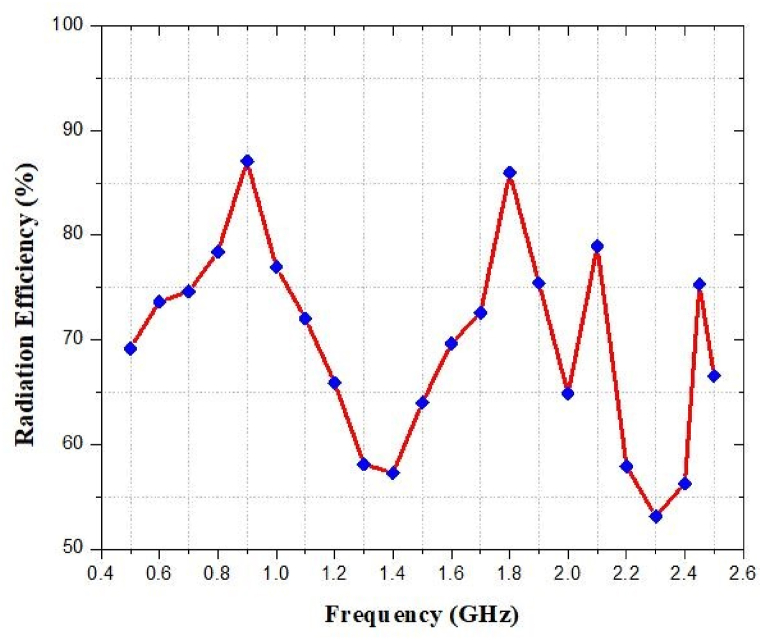
Efficiency against input power of the proposed wireless charging system.

Figure-44 illustrates the simulated radiation efficiency at a variety of various frequencies. The antenna radiation efficiency is 87% at 0.9 GHz, 86% at 1.8 GHz, 78% at 2.1 GHz and 75% at 2.45 GHz. Simulated results illustrate that an antenna can successfully send and receive radio frequency signals at its design frequencies.

### 6.9 Return loss against bending and twisting

**Fig. 45:**
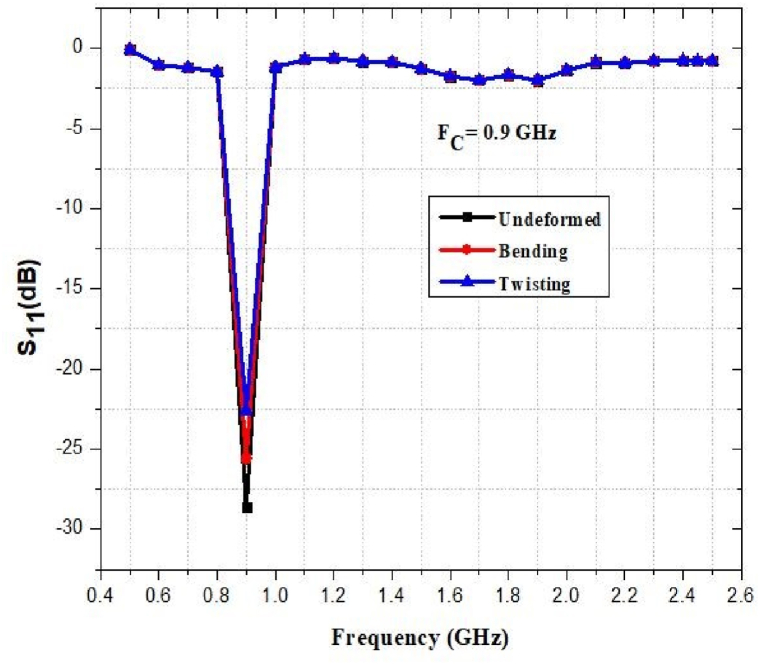
Return loss at 0.9 GHz.

**Fig. 46:**
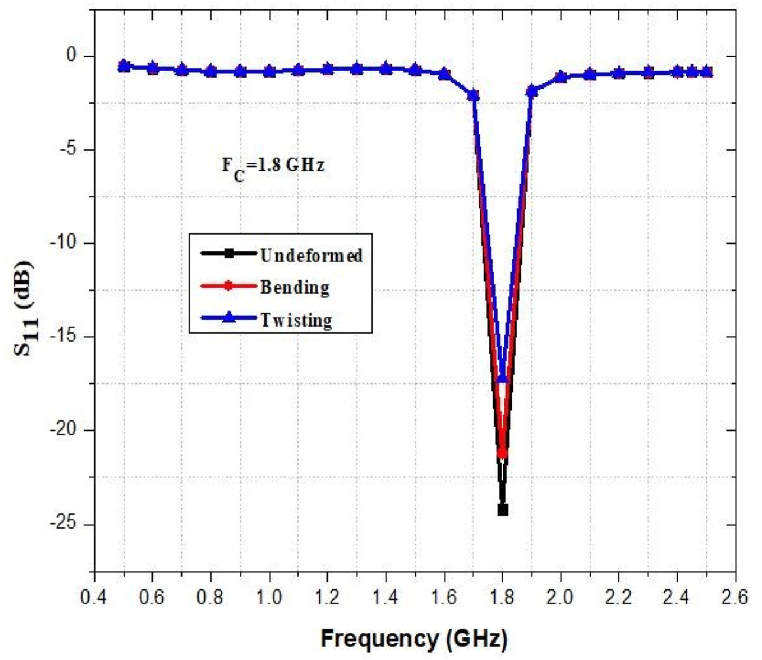
Return loss at 1.8 GHz.

**Fig. 47:**
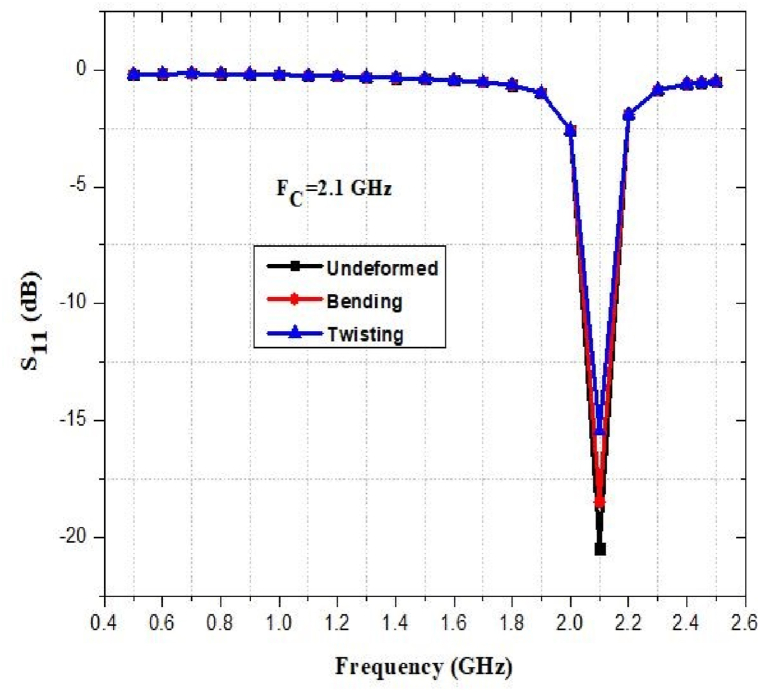
Return loss at 2.1 GHz.

**Fig. 48:**
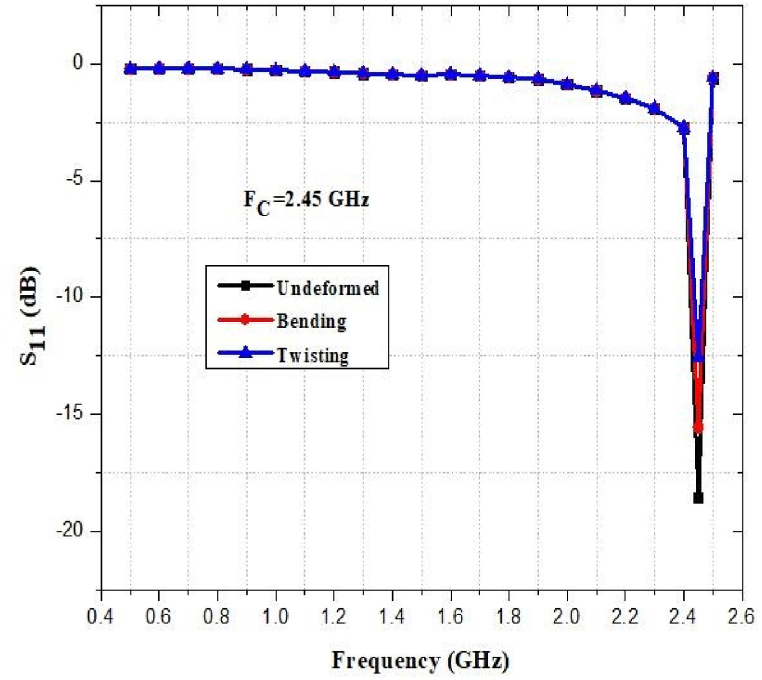
Return loss at 2.45 GHz.

The flexibility and stretchability of the antenna have been evaluated in terms of its performance. Fig. 45, Fig. 46, Fig. 47, Fig. 48 show how the return loss of the designed antenna has shifted or decreased as a result of bending, and twisting at different frequencies. As seen, the resonant frequency is quite steady, with a maximum frequency decrease of approximately 1% and 2% for bending and twisting respectively.

### 6.10 Radiation efficiency against bending and twisting

**Fig. 49:**
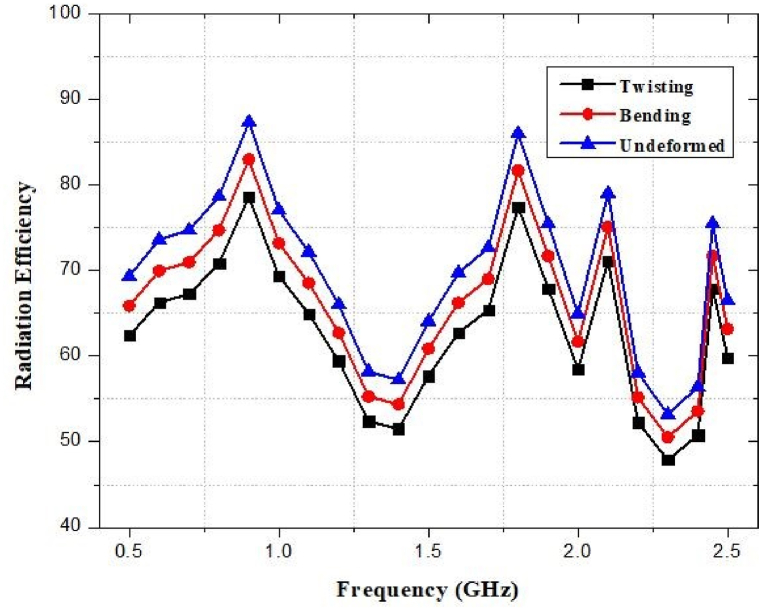
Response of radiation efficiency under bending and twisting.

Similar outcomes are observed in terms of total efficiency, where the variance range is approximately 5% and 10% respectively, for bending and twisting. Fig. 49 depicts the simulated radiation behavior of the designed antenna that has a substantial impact on the radiation behavior owing to bending and twisting. However, the overall form of the radiation pattern does not change.

### 6.11 Gain against bending and twisting

**Fig. 50:**
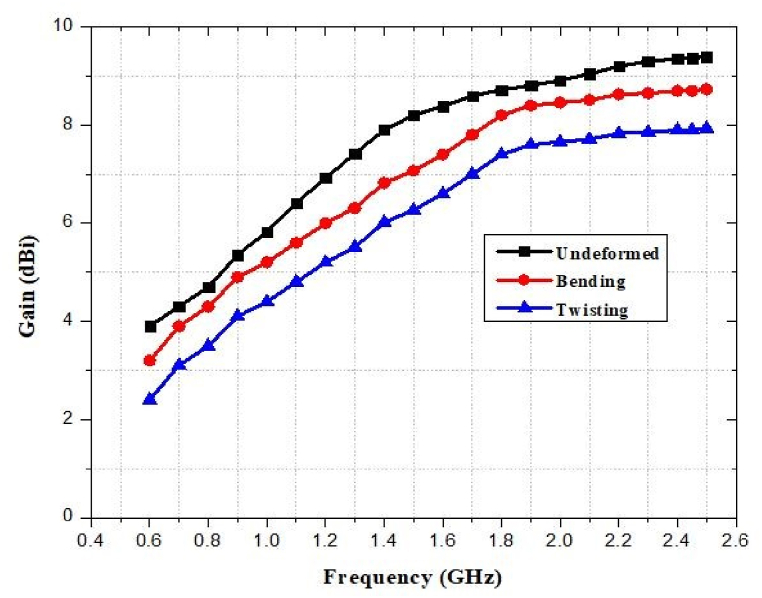
Response of the reported antenna gain under bending and twisting.

The gain predicted for various radii of curvature and twisting is presented. As the curvature rises, the back radiation also increases, resulting in a decrease in the antenna gain. As seen, the gain is quite steady, with a maximum gain decrease of approximately 2% and 4% for bending and twisting respectively. Fig. 50 depicts the simulated gain of the designed antenna that has a substantial impact on bending and twisting. However, the overall form of the radiation pattern does not change.

### 6.12 Response of resistivity under stretching

**Fig. 51:**
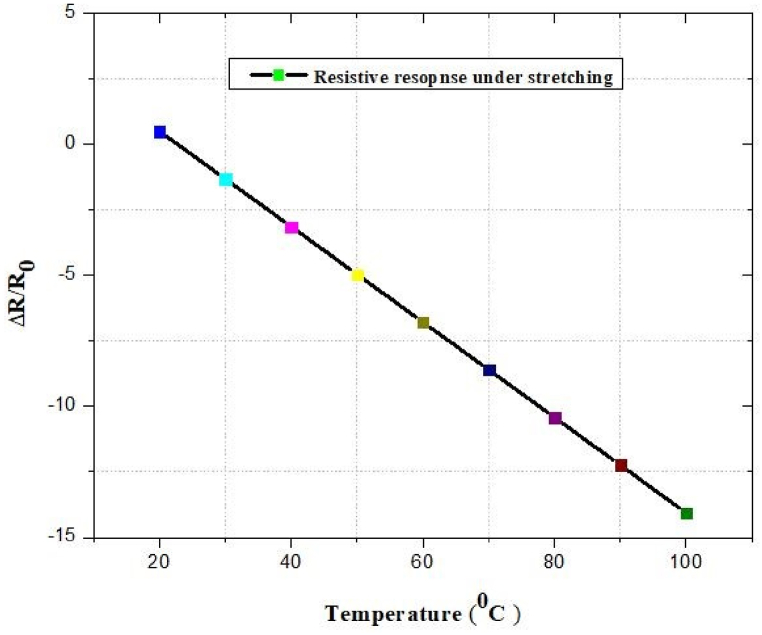
Simulated resistive response of the LIG temperature sensor under stretching.

The simulation result explores the effect of nanosecond and picosecond laser fluence and scanning speed on the selective removal of FR4 from the substrate. Using the FEM simulations tool, both the heat generated during laser ablation and the stress distributions in the thin layer have been investigated. Fig. 51 depicts the response of resistivity of the designed antenna at different temperature, such as for temperatures between 0^*o*^C and 100^*o*^C, the star-shaped trace-patterned LIG temperature sensor is both sensitive and linear.

### 6.13 Resistive response against inward and outward bending

**Fig. 52:**
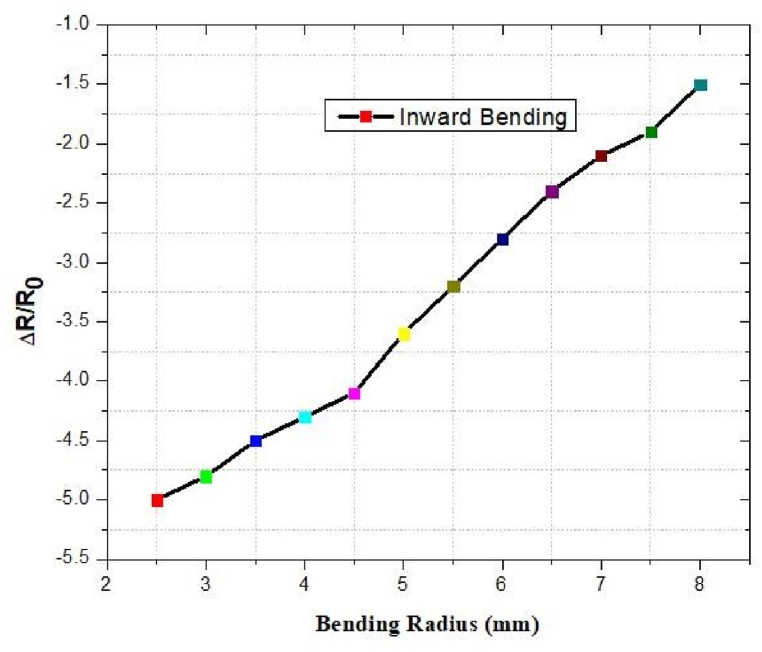
Inward bending.

**Fig. 53:**
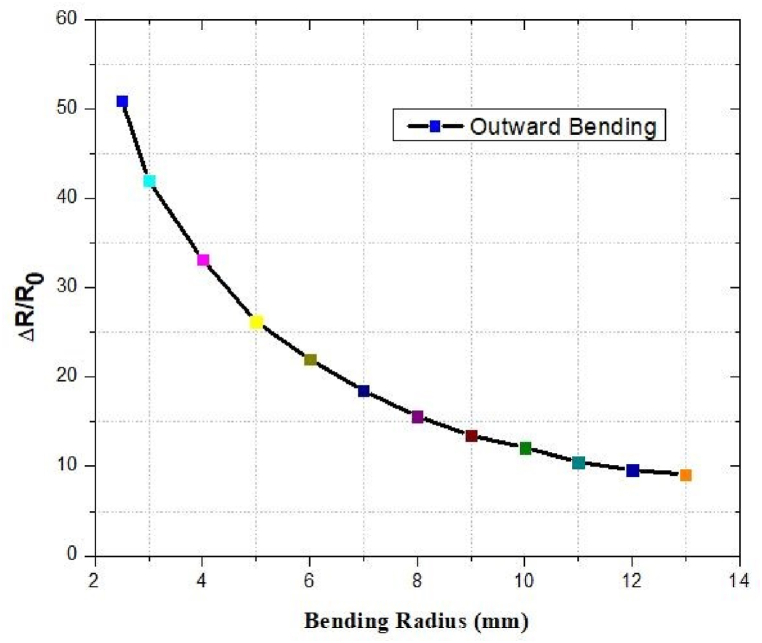
Outward bending.

The outcomes of the reported antenna under bending conditions are depicted in Fig. 52 and Fig. 53. In the inward bending test, Δ*R/R*_0_ increased to as the radius of bending decreased. The resistance did not continue to decrease when we decreased the radius of curvature. The resistance drop was caused by the overlap of the crumpled graphene layers. In the outward bending test, as the bending radius reduced, Δ*R/R*_0_ increased.

### 6.14 Strain sensor under stretching mode (various frequency conditions)

**Fig. 54:**
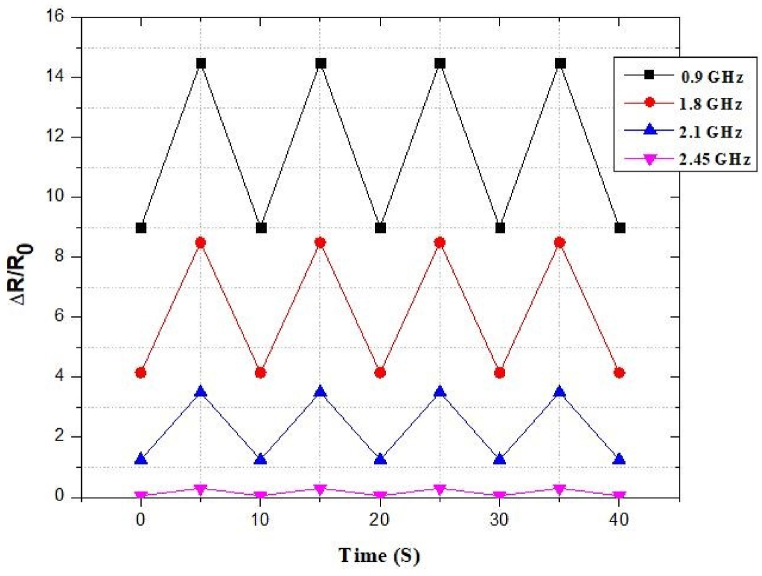
Response of resistivity under stretching at different frequencies.

Fig. 54 illustrates the fluctuation of the resistance at different frequencies. The curves exhibited excellent stability. It also shows the relationship between Δ*R/R*_0_ and time at 0.9 GHz, 1.8 GHz, 2.1 GHz, 2.45 GHz respectively.

### 6.15 Strain sensor under stretching mode (various strain conditions)

**Fig. 55:**
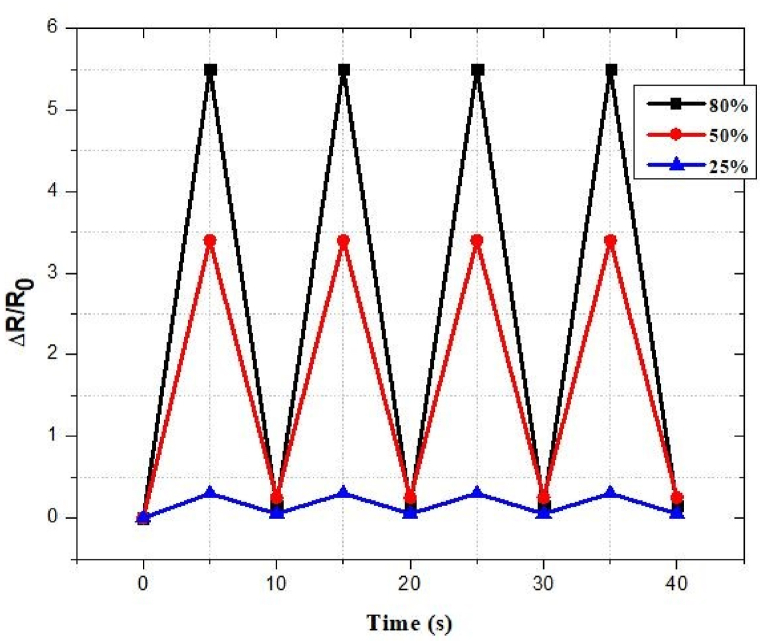
Response of the proposed strain-sensored antenna to different strain situations.

Fig. 55 demonstrates the simulated features of the reported strain sensor antenna. We performed several loading–unloading simulations at 25%, 50%, and 80%, respectively, tensile stresses. It also shows the relationship between Δ*R/R*_0_. In addition to measuring EEG signals, stretchy electrodes based on a star-shaped mesh structure can be used.

### 6.16 Response of sensitivity related to humidity

**Fig. 56:**
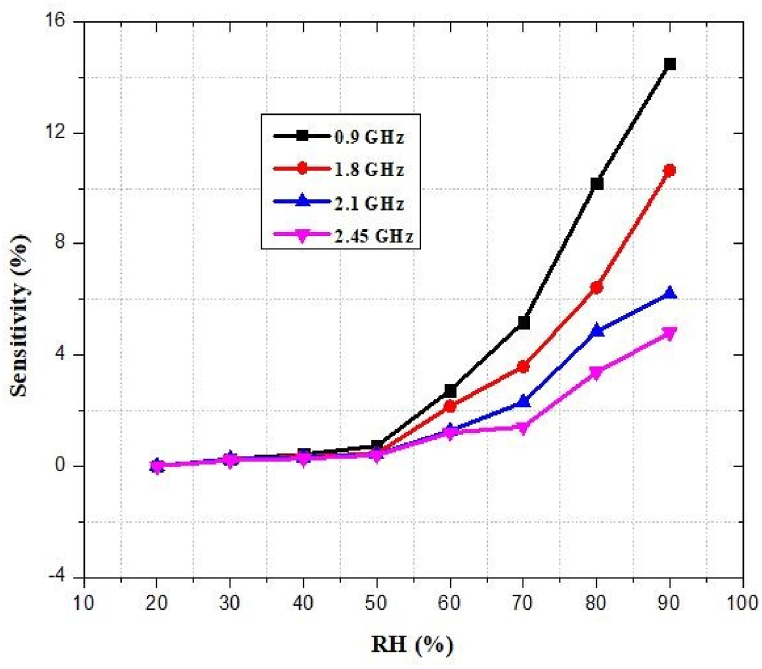
Capacitive response of the LIG hydration sensor with star-shaped configuration in different relative humidity (RH).

Fig. 56 depicts the sensitivity response of the designed antenna at different RH. As the relative humidity rises, a star-shaped humid sensor exhibits a frequency-dependent increase in measured capacitance. The temperature and humidity sensors are reliable and robust due to their winding and spiral structures that resist mechanical deformations. The resistance-based temperature sensor demonstrates almost no variation upon stretching, indicating robust and reliable functioning despite deformations. Capacitance changes by only 1.2% when the humidity sensor is stretched by 20%, corroborating previous reports of sensor stability even after deformations.

### 6.17 Conversion efficiency against input power

**Fig. 57:**
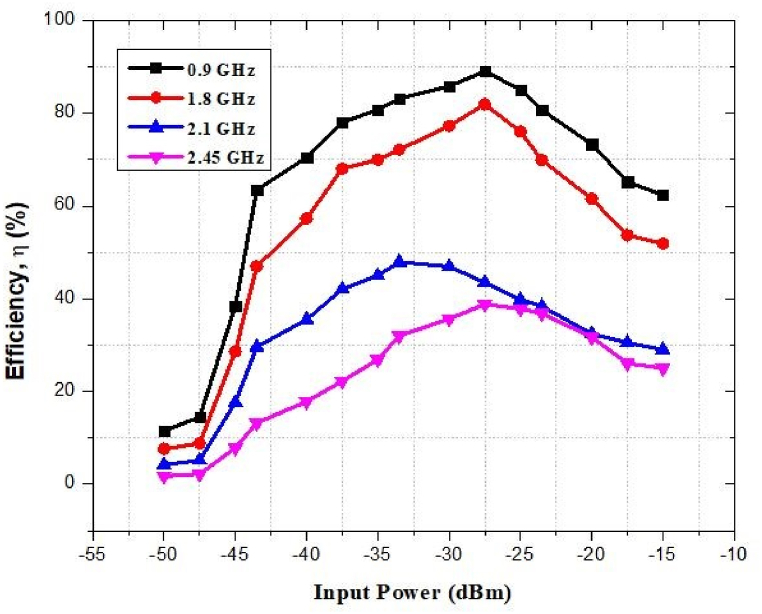
Efficiency against input power of the proposed wireless charging system.

The efficiency of the rectifier circuit at 0.9 GHz, 1.8 GHz, 2.1 GHz, and 2.45 GHz is shown in Fig. 57. The highest efficiency of the rectifier is almost 90%, 80% and 40% when it obtains RF power at just −27.5 dBm at 0.9 GHz, 1.8 GHz and 2.45 GHz respectively. It is 48% for the 2.1 GHz frequency band when input power is −35 dBm.

### 6.18 Output voltage against multiple RF signal

**Fig. 58:**
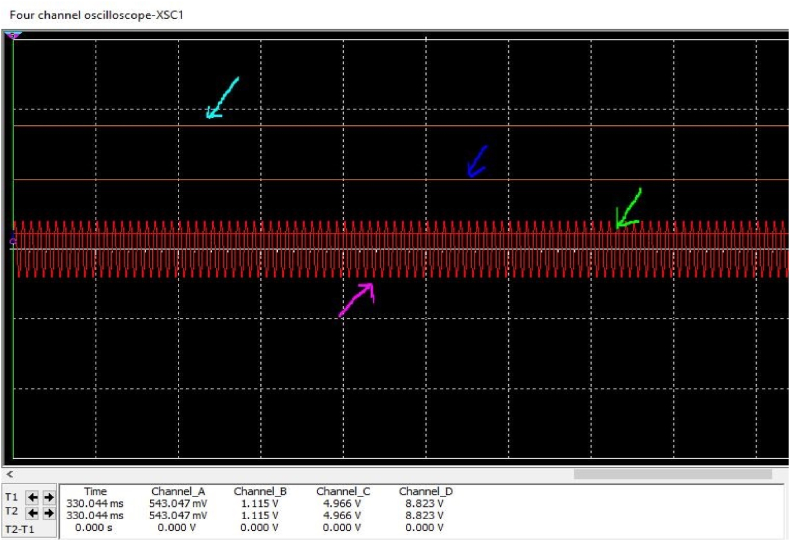
Output voltage against received multiple RF signal.

Fig. 58 depicts the output voltage of the proposed antenna for a multiband rectenna system. The aqua-colored arrow shows that the output voltage is a combination of 0.9, 1.8, 2.1, and 2.45 GHz signals. The blue arrow represents 1.8 GHz, 2.1 GHz, and 2.45 GHz signals in the output voltage. The lime-colored arrow shows the output RF/DC voltage of 2.1 GHz and 2.45 GHz signals. This method uses a 4-stage voltage multiplier.

### 6.19 Output voltage against input power

**Fig. 59:**
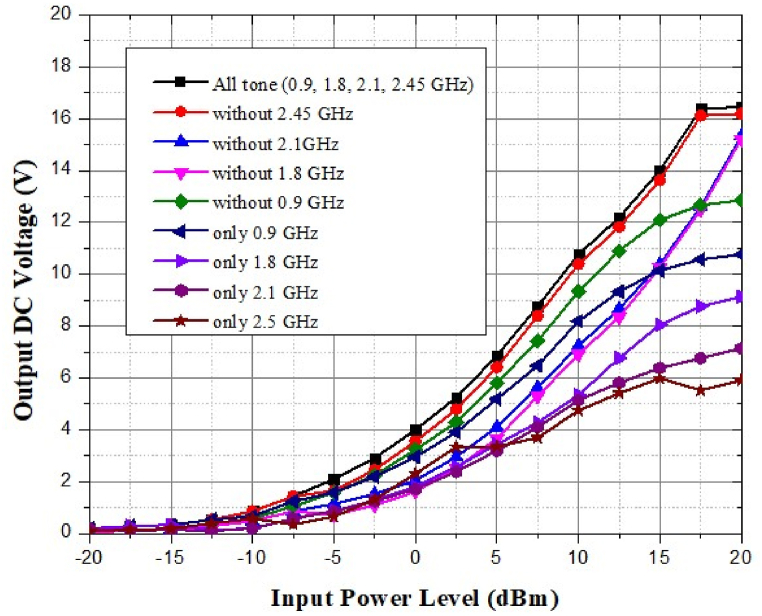
Simulated output voltage against input power.

Fig. 59 shows the simulated output voltage of the proposed antenna for a multi-band receiver system against input power from −20 dB to 20 dB. The discrete output voltage is highest at 0.9 GHz and gradually decreases to the minimum at 2.45 GHz. All frequency ranges have maximum integrated output voltage. As frequencies go away, the output voltage value drops from 0.9 GHz to 2.45 GHz.

### 6.20 Open circuit voltage and voltage conversion efficiency (OCV) against number of stages

**Fig. 60:**
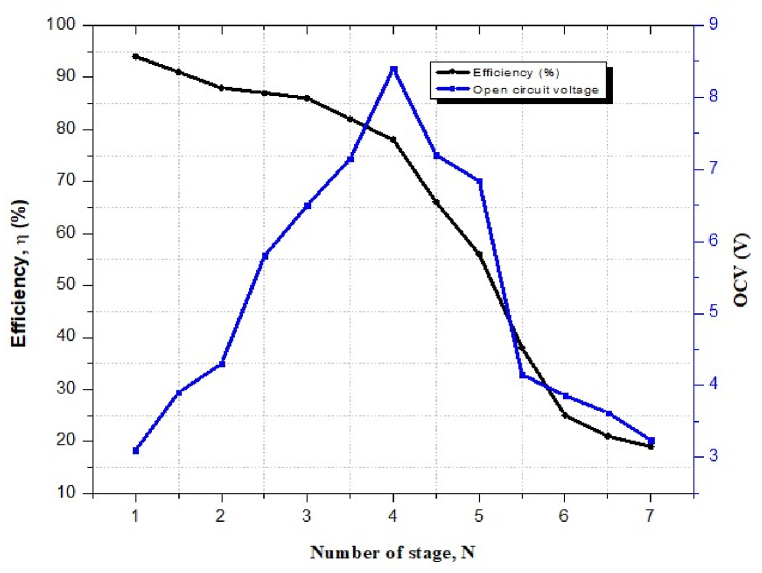
Simulated OCV and VCE versus the number of cascaded stages.

As shown in Fig. 60, the open circuit voltage (OCV) and VCE of the RBR are meant to replicate an input power from the antenna that is approximately 10 dBm in level. These levels can be selected independently. At first, as the boosting phases go on, the OCV goes up, but the VCE goes down by a lot. When the number of boosting steps reaches 4, the OCV declines and the VCE falls. This is the best outcome of our design. To reach almost 8.3 V OCV and 94% percent VCE, a 4-stage RBR is used, resulting in a voltage boost of 3.6.

### 6.21 Open circuit voltage (OCV) of the proposed method against distance

**Fig. 61:**
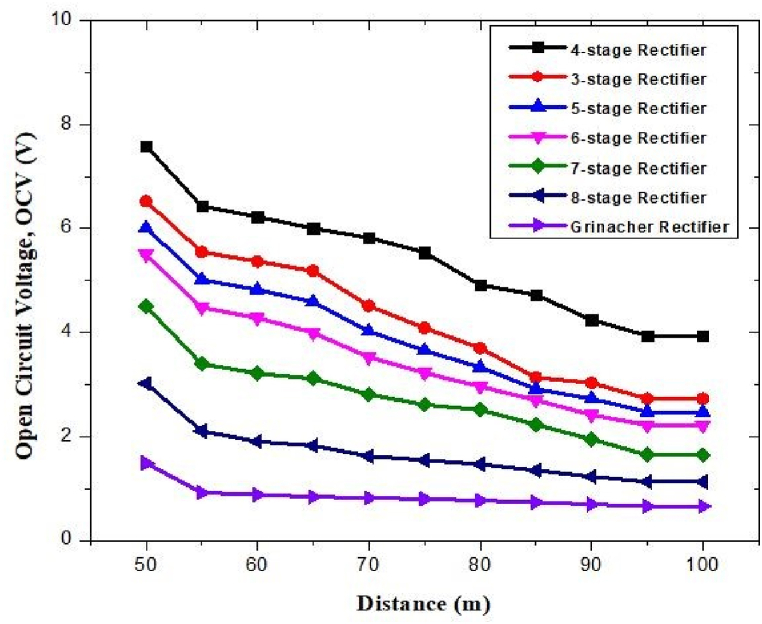
The simulated OCV, along with that of the Greinacher rectifier, was obtained by plotting against the number of stages.

Fig. 61 shows our design includes both a Greinacher rectifier and RBR energy harvesters with 3, 4, 5, 6, 7 and 8 stages, respectively. The observed OCV data versus distance show that our circuit outperforms three others. The proposed system is capable of delivering an output voltage of almost 5 V even when there is a separation of 100 cm between both the antenna and the rectenna. In comparison to the Greinacher rectifier, the suggested rectifier is capable of producing output voltage that is up to five times greater. This is produced by an extra inductor, sister circuit and low-pass filter.

### 6.22 Output voltage of the proposed circuit system

**Fig. 62:**
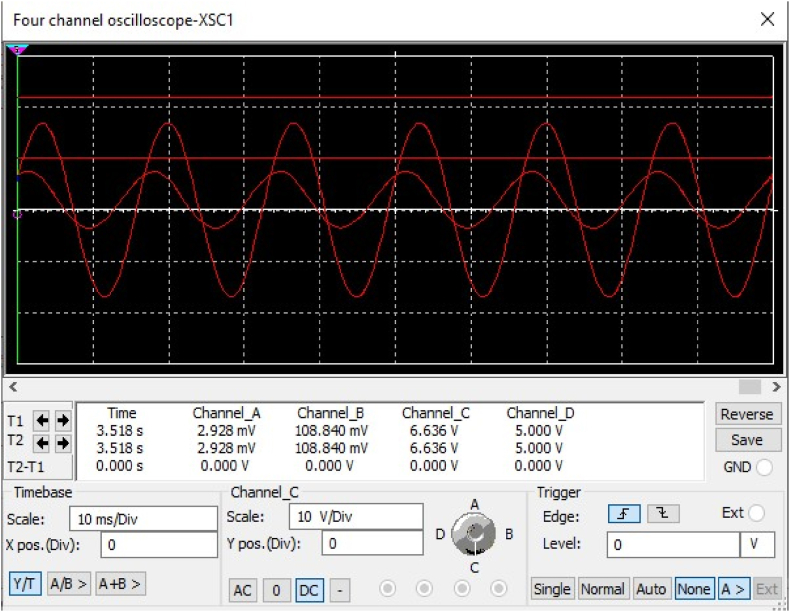
Final output of the proposed wireless charging system.

In order to charge a mobile device, the received RF power must first be amplified by the voltage multiplier circuit. Depending on the input power, the RF/DC voltage multiplier's output voltage fluctuates. As a result, HSMS-2822 and HSMS-2852 based two sister circuit is concerned. Depending on the distance between Tx and Rx, the output voltage may vary. However, the phone requires a regular stream of electricity in order to charge its battery. A supercapacitor is used in the device as an assortment to charge the phone at any time. Channels A, B, C, and D, in Fig. 62 show the incident RF signals, the rectified voltage after impedance matching, the output voltage of the voltage multiplier, and the final output voltage of the voltage regulator, respectively.

### 6.23 Useable harvested energy against capacitor size

**Fig. 63:**
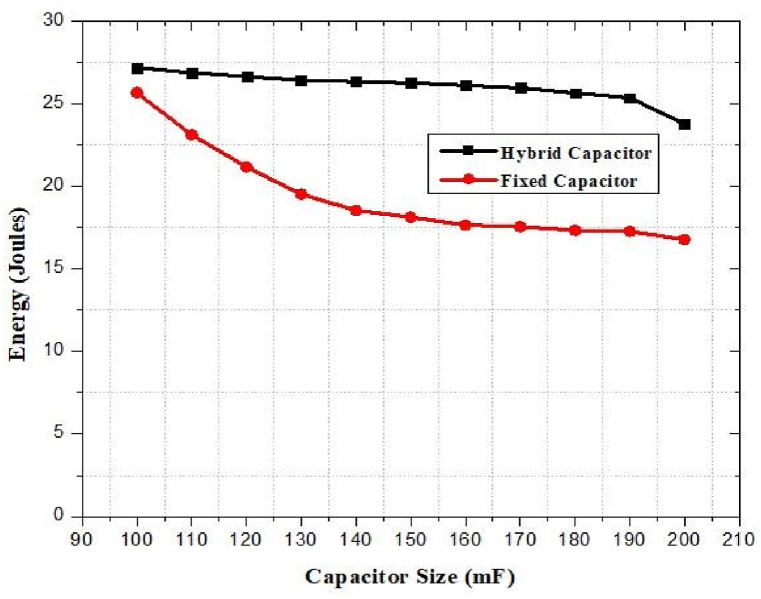
The amount of useable harvested energy in the fixed and hybrid capacitor configurations.

The simulation result illustrates how the capacitance influences the overall useful gathered energy. As can be seen in Fig. 63, a rise in capacitor size from 100 mF to 200 mF results in a 42% drop in useable harvested energy. One 100 mF capacitor and one larger capacitor in the 100–200 mF range made up the hybrid design. The hybrid approach maintains its performance across a wide variety of capacitor sizes.

### 6.24 Charging area coverage in relation to capacitor size

**Fig. 64:**
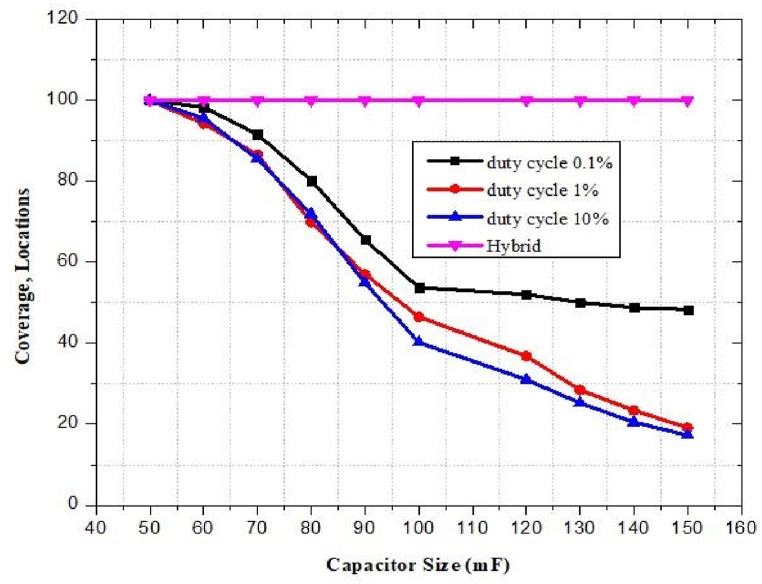
Coverage of geographical area in relation to capacitor size for charging the mobile phone.

The proposed method exhibits constant performance for diverse capacitor sizes. Maximum coverage is attained with the lowest duty cycle for a fixed capacitor configuration is shown in Fig. 64. Increasing the size of the fixed capacitor from 100 mF to 200 mF reduces the coverage area by 45% and 80% at duty cycles of 0.1% and 1% respectively. The hybrid arrangement gives consistent coverage of close to 100% for all capacitor sizes. Hybrid setup performance is independent of larger capacitor size because the switch is only made when enough energy is available to charge the larger capacitor.

### 6.25 Activations of stored energy in relation to capacitor size

**Fig. 65:**
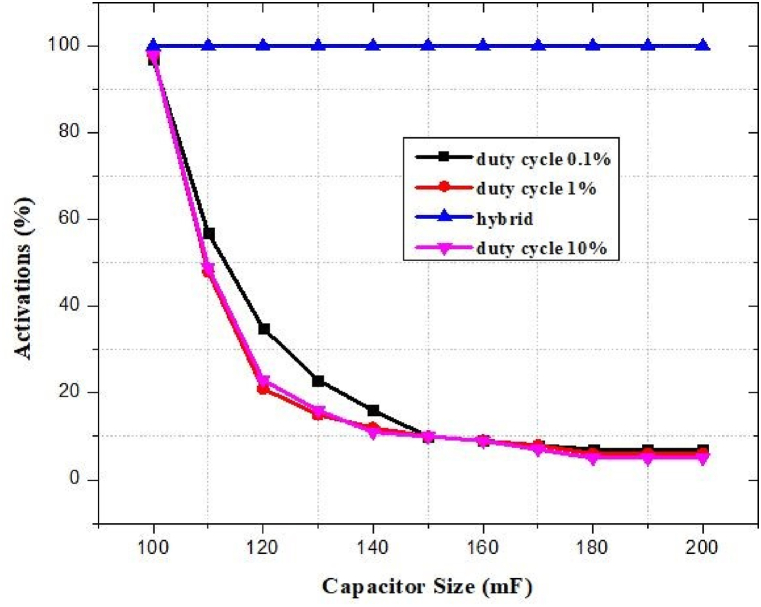
Stored energy activation in relation to capacitor size for charging the mobile phone.

Fig. 65 depicts the activations of stored energy in relation to capacitor size. It is evident that the size of a capacitor has a significant impact on stored energy activation, decreasing it by up to 92%. The hybrid setup is unaffected by the size of the capacitor and reliably achieves 100% activation. Since the switch is only performed when there is enough energy to charge the larger capacitor, the number of sensor activation for a hybrid setup is independent of the larger capacitor size.

### 6.26 Charging time against distance

**Fig. 66:**
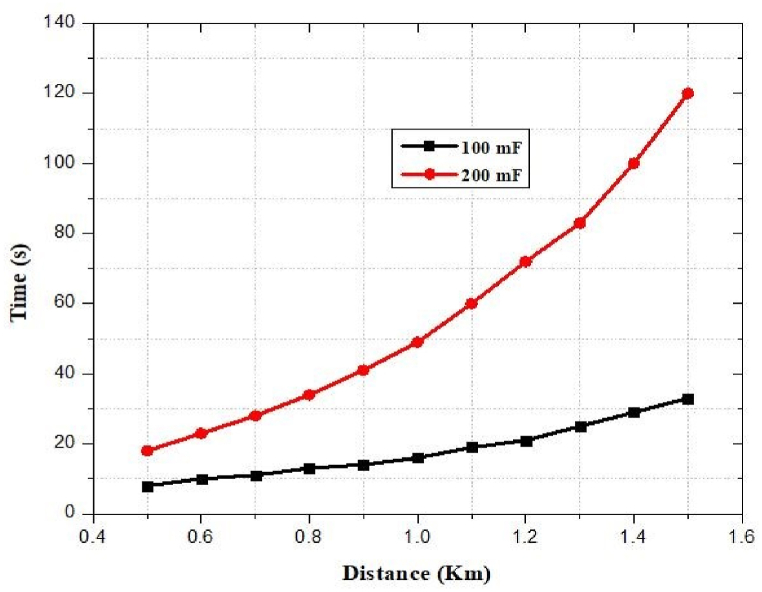
Charging Time in relation to capacitor size of the storage device.

Fig. 66 depicts the charging time in relation to the distance of the energy harvesting network from the RF station. Charging time for super-capacitors with capacities of 100 mF and 200 mF rises exponentially with distance.

### 6.27 Life time of the charging system

**Fig. 67:**
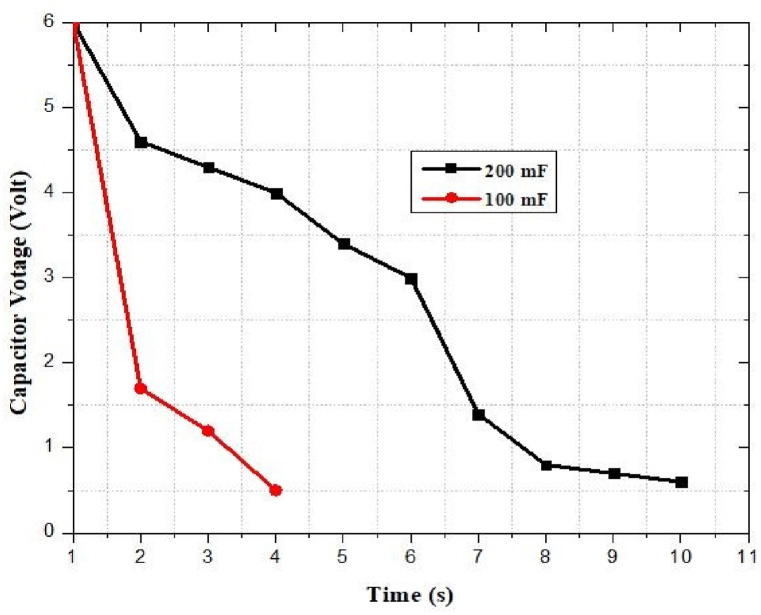
Charging Time in relation to capacitor size of the storage device.

Fig. 67 depicts the lifetime of charging system that executes a trivial application and periodically wakes up to measure temperature before going to sleep again for 10 s. With a 100 mF capacitor, the longest sensor lifetime was 4 s, whereas with a 200 mF capacitor, it was 10 s.

### 6.28 Internal resistance of the mobile phone battery (Li-pro)

**Fig. 68:**
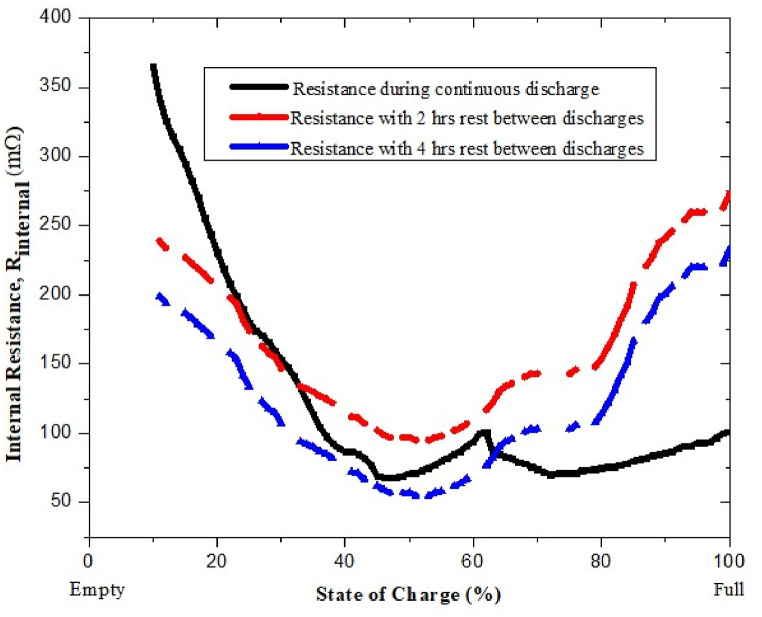
Internal resistance versus state of charge.

**Fig. 69:**
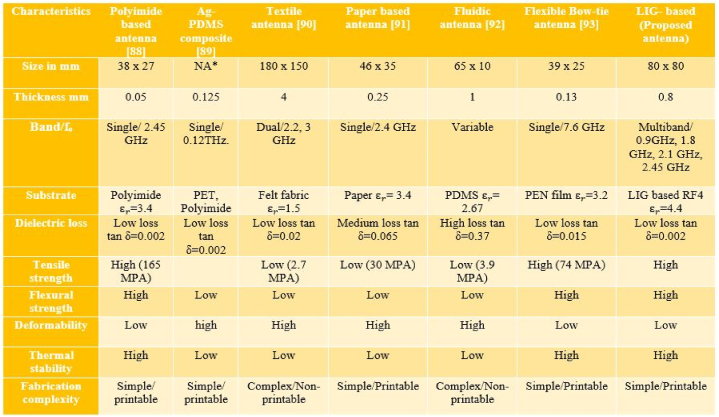
Comparison of the proposed flexible and stretchable antenna with related antennas.

Fig. 68 shows the internal resistance of the mobile phone battery during charging and discharging. The internal resistance of a battery fluctuates depending on its level of charge. The Li-Pro-based batteries show the most significant modifications. The internal resistance of Li-Pro is shown in Fig. 15 while it is empty, during charge, at full charge, and after a 2-h and 4 h rest period. Resistance is strongest at low charge states and soon after charging. The internal battery resistance lowers during discharge, reaches its lowest point at 50% charge, andthen starts creeping up again (dotted line) (see Fig. 69).

**Fig. 70:**
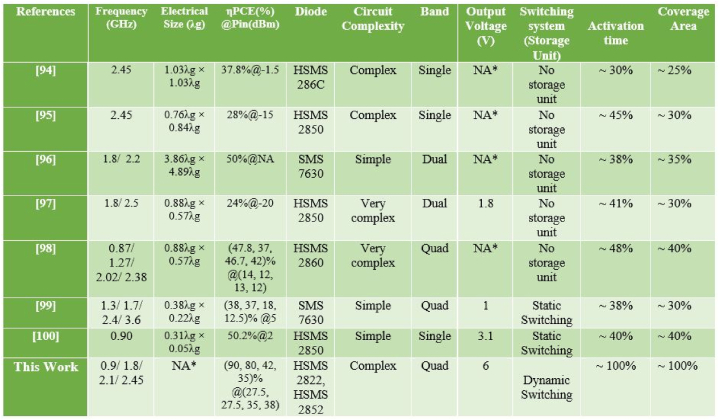
Comparison of the proposed rectifier and related.

## 7 Conclusion

This study focuses on the design and development of a reconfigurable multiband antenna that operates at frequencies of 0.9 MHz, 1.8 GHz, 2.1 GHz, and 2.45 GHz. This frequency is suitable for radio frequency harvesting devices. In contrast to previous efforts, we use spectrum sensing as a preprocessing step to identify the frequency at which the maximum power exceeds a predetermined threshold. After filtering, a voltage multiplier circuit based on HSMS-2822 and HSMS-2852 receives this signal with maximum power. To provide smooth DC voltage, a low-pass filter is used in the proposed multiplier circuit. With 0 dbm of RF input power, the highest efficiency is 80% and the output voltage of the model is 8.8 V. It indicates that receiving RF energy from multiple bands simultaneously enhances the received power more competitive. The simulated results depict that the suggested antenna is capable of producing outstanding outcomes.

## Author contribution statement

Pankaj Chandra Kar: Conceived and designed the experiments; Performed the experiments; Analyzed and interpreted the data; Contributed reagents, materials, analysis tools or data; Wrote the paper.

Md. Ariful Islam: Conceived and designed the experiments; Analyzed and interpreted the data; Wrote the paper.

## Funding statement

This research did not receive any specific grant from funding agencies in the public, commercial, or not-for-profit sectors.

## Data availability statement

Data will be made available on request.

## Declaration of interest's statement

The authors declare no competing interests.

## References

[1] Piñuela M., Mitcheson P.D., Lucyszyn S. (July 2013). Ambient RF energy harvesting in urban and semi-urban environments. IEEE Trans. Microw. Theor. Tech..

[2] Bouchouicha D., Dupont F., Latrach M., Ventura L. (2010, March). Ambient RF energy harvesting. Int. Conf. Renewable Energ. Power Quality.

[3] Krishnamoothy R., Umapathy K. (2018). 2018 Fourth International Conference on Advances in Electrical, Electronics, Information, Communication and Bio-Informatics.

[4] Palazzi V. (Jan. 2018). A novel ultra-lightweight multiband rectenna on paper for RF energy harvesting in the next generation LTE bands. IEEE Trans. Microw. Theor. Tech..

[5] Muncuk U., Alemdar K., Sarode J.D., Chowdhury K.R. (2018). Multiband ambient RF energy harvesting circuit design for enabling batteryless sensors and IoT. IEEE Internet Things J..

[6] Ahmad A., Alam M.S., Chabaan R. (March 2018). A comprehensive review of wireless charging technologies for electric vehicles. IEEE Trans. Transport. Electrification.

[7] Brown William C. (1969). IEEE Transactions on Aerospace and Electronic Systems AES-5.

[8] Tesla N. (1904).

[9] Sengupta D.L., Sarkar T.K., Maxwell Hertz (April 2003). The Maxwellians, and the early history of electromagnetic waves. IEEE Antenn. Propag. Mag..

[10] Kurs A., Karalis A., Moffatt R., Joannopoulos J.D., Fisher P., Soljacic M. (2007). Wireless power transfer via strongly coupled magnetic resonances. Science.

[11] Chen Q., Yuan Q. (2016). Handbook of Antenna Technologies.

[12] Lu Xiao (2015). Wireless charging technologies: fundamentals, standards, and network applications. IEEE Commun. Surveys Tutorials.

[13] Chen Z., Xi J., Huang W., Yuen M.M. (2017). Stretchable conductive elastomer for wireless wearable communication applications. Sci. Rep..

[14] Rai T., Dantes P., Bahreyni B., Kim W.S. (2013). A stretchable RF antenna with silver nanowires. IEEE Electron. Device Lett..

[15] Song L., Myers A.C., Adams J.J., Zhu Y. (2014). Stretchable and reversibly deformable radio frequency antennas based on silver nanowires. ACS Appl. Mater. Interfaces.

[16] Zhu J., Fox J.J., Yi N., Cheng H. (2019). Structural design for stretchable microstrip antennas. ACS Appl. Mater. Interfaces.

[17] Wang C., Takei K., Takahashi T., Javey A. (2013). Carbon nanotube electronics moving forward. Chem. Soc. Rev..

[18] Ye R., James D.K., Tour J.M. (Jan. 2019). Laser-induced graphene: from discovery to translation. Adv. Mater..

[19] Sun B., McCay R.N., Goswami S., Xu Y., Zhang C., Ling Y., Lin J., Yan Z. (2018 Dec). Gas-permeable, multifunctional on-skin electronics based on laser-induced porous graphene and sugar-templated elastomer sponges. Adv. Mater..

[20] Lipomi D., Vosgueritchian M., Tee B.K. (2011). Skin-like pressure and strain sensors based on transparent elastic films of carbon nanotubes. Nat. Nanotechnol..

[21] Cheng Y., Wang R., Sun J., Gao L. (2015). A stretchable and highly sensitive graphene based fiber for sensing tensile strain, bending, and torsion. Adv. Mater..

[22] Jia Zhu, Hu Zhihui, Song Chaoyun, Ning Yi, Yu Zhaozheng, Liu Zhendong, Liu Shangbin, Wang Mengjun, Gregory Dexheimer Michael, Yang Jian, Cheng Huanyu (2021). Stretchable wideband dipole antennas and rectennas for RF energy harvesting. Materials Today Physics.

[23] Bougas I.D., Papadopoulou M.S., Boursianis A.D., Kokkinidis K., Goudos S.K. (2021). State-of-the-Art techniques in RF energy harvesting circuits. Tele.com (NY).

[24] Pavone D., Buonanno A., D'Urso M., Della Corte F. (2012). Design considerations for radio frequency energy harvesting devices. Prog. Electromagnetics Res. B.

[25] Fan S. (2018).

[26] Nintanavongsa P., Muncuk U., Lewis D.R., Chowdhury K.R. (March 2012). Design optimization and implementation for RF energy harvesting circuits. IEEE J. Emerg. Selected Topics in Circuits and Systems.

[27] Zeng M., Andrenko A.S., Liu X., Li Z., Tan H.-Z. (2017). A compact fractal loop rectenna for RF energy harvesting. IEEE Antenn. Wireless Propag. Lett..

[28] Sun H., Guo Y., He M., Zhong Z. (2012). Design of a high-efficiency 2.45-GHz rectenna for low-input-power energy harvesting. IEEE Antenn. Wireless Propag. Lett..

[29] Strassner B., Chang Kai (June 2003). Highly efficient C-band circularly polarized rectifying antenna array for wireless microwave power transmission. IEEE Trans. Antenn. Propag..

[30] Nintanavongsa Prusayon, A Survey on RF Energy Harvesting (2014). Circuits and protocols. Energy Proc..

[31] Rauniyar A., Engelstad P., Østerbø O.N. (2018). RF energy harvesting and information transmission based on NOMA for wireless powered IoT relay systems. Sensors.

[32] Balanis C.A. (2015).

[33] Akin-Ponnle A.E., Carvalho N.B. (2021). Energy harvesting mechanisms in a smart city—a review. Smart Cities.

[34] Erol-Kantarci M., Illig D., Rumbaugh L., Jemison W. (2017). Cyber Physical Systems.

[35] Ibrahim H.H., Singh M.J., Al-Bawri S.S., Ibrahim S.K., Islam M.T., Alzamil A., Islam M.S. (2022). Radio frequency energy harvesting technologies: a comprehensive review on designing, methodologies, and potential applications. Sensors.

[36] Zakaria Zahriladha (2015). Applied Mechanics and Materials.

[37] Krishnamoorthy R., Kumar N., Grebennikov A., Ramiah H. (2018). A high-efficiency ultra-broadband mixed-mode GaN HEMT power amplifier. IEEE Trans. Circuits and Systems II: Express Briefs.

[38] Singh A.K., Raman A. (2018). 2018 2nd International Conference on Trends in Electronics and Informatics.

[39] Yeole D.S., Khot U.P. (2016). IEEE International Conference on Recent Trends in Electronics.

[40] Pattapu Udayabhaskar, Das Sushrut (2018). 2018 IEEE Indian Conference on Antennas and Propogation (InCAP). IEEE.

[41] Takhedmit Hakim (2010). A 2.45-GHz dual-diode RF-to-dc rectifier for rectenna applications. *The 40th European Microwave Conference*. IEEE.

[42] Yang Kansheng (2016). A coplanar vivaldi antenna with integrated filter for ka-band. 2016 loughborough antennas propagation conference (LAPC). IEEE.

[43] Yang Kansheng (2018). Dual-stub Ka-band Vivaldi antenna with integrated bandpass filter. IET Microw., Antennas Propag..

[44] Nurhayati Nurhayati, Setijadi Eko, Hendrantoro Gamantyo (2019). Radiation pattern analysis and modelling of coplanar vivaldi antenna element for linear array pattern evaluation. Prog. Electromag. Res. B.

[45] Zhou Jian, Xu Xuezhu, Yu Hu, Lubineau Gilles (2017). Deformable and wearable carbon nanotube microwire-based sensors for ultrasensitive monitoring of strain, pressure and torsion. Nanoscale.

[46] Cheng S., Rydberg A., Hjort K., Wu Z. (2009). Liquid metal stretchable unbalanced loop antenna. Appl. Phys. Lett..

[47] Nikbakhtnasrabadi Fatemeh (2021). Textile-based stretchable microstrip antenna with intrinsic strain sensing. ACS Appl. Electronic Mat..

[48] Bhattacharjee M., Nikbakhtnasrabadi F., Dahiya R. (2021). Printed chipless antenna as flexible temperature sensor. IEEE Internet Things J..

[49] Escobedo P., Bhattacharjee M., Nikbakhtnasrabadi F., Dahiya R. (2021). Smart bandage with wireless strain and temperature sensors and batteryless NFC tag. IEEE Internet Things J..

[50] Muhammad Surajo Tiang, Wong Jun-Jiat, Iqbal Sew-Kin, Ernesto Mohammad Limiti, Amjad & Alibakhshikenari (2020). Compact rectifier circuit design for harvesting GSM/900 ambient energy. Electronics.

[51] Dang W., Vinciguerra V., Lorenzelli L., Dahiya R. (2017). Printable stretchable interconnects. Flex. Print Electron..

[52] Kubo M., Li X., Kim C., Hashimoto M., Wiley B.J., Ham D., Whitesides G.M. (2010). Stretchable microfluidic radiofrequency antennas. Adv. Mater..

[53] Tata U., Huang H., Carter R., Chiao J. (2009). Exploiting a patch antenna for strain measurements. Meas. Sci. Technol..

[54] Pham B.L., Pham A. (2013).

[55] Kar P.C., Islam M.A., Paul A., Chakrabarti J., Sutradhar S., Rahman F.T. (2022). Experimental antenna and circuit model for charging an electronic device from the ambient electromagnetic waves. Am. J. Eng. Appl. Sci..

[56] Hsu C.-Y., Lin S.-C., Tsai Z.-M. (July 2017). Quadband rectifier using resonant matching networks for enhanced harvesting capability. IEEE Microw. Wireless Compon. Lett..

[57] Al-Azawy M.M., Sari F. (2019). 2019 1st Global Power, Energy and Communication Conference.

[58] Ibrahim H.H., Singh M.J., Al-Bawri S.S., Ibrahim S.K., Islam M.T., Alzamil A., Islam M.S. (2022). Radio frequency energy harvesting technologies: a comprehensive review on designing, methodologies, and potential applications. Sensors.

[59] Sherazi Husnain, Zorbas Dimitrios, O'Flynn Brendan (2022). A comprehensive survey on RF energy harvesting: applications and performance determinants. Sensors.

[60] Lee T.H. (2003).

[61] Tran Hung, Akerberg Johan, Björkman Mats, Tran Ha-Vu (2019). RF energy harvesting: an analysis of wireless sensor networks for reliable communication. Wireless Network.

[62] Khaleel H.R., Al-Rizzo H.M., Abbosh A.I. (2013). Advancement in Microstrip Antennas with Recent Applications.

[63] Mohanta Harish, Abbas Chandra Kouzani, Mandal Sushanta (2019). Reconfigurable antennas and their applications. Univ. J. Elect. Electron. Eng..

[64] Nikbakhtnasrabadi Fatemeh, El Matbouly Hatem, Ntagios Markellos, Dahiya Ravinder (2021). ACS Appl. Electron. Mat..

[65] Jia Zhu, Hu Zhihui, Song Chaoyun, Ning Yi, Yu Zhaozheng, Liu Zhendong, Liu Shangbin, Wang Mengjun, Gregory Dexheimer Michael, Yang Jian, Cheng Huanyu (2021). Stretchable wideband dipole antennas and rectennas for RF energy harvesting. Materials Today Physics.

[66] Khaleel Haider (2012).

[67] Leng T., Huang X., Chang K., Chen J., Abdalla M.A., Hu Z. (2016). Graphene nanoflakes printed flexible meandered-line dipole antenna on paper substrate for low-cost RFID and sensing applications. IEEE Antenn. Wireless Propag. Lett..

[68] Paracha K.N., Rahim S.K.A., Chattha H.T., Aljaafreh S.S., Ur Rehman S., Lo Y.C. (2018). Low-cost printed flexible antenna by using an office printer for conformal applications. Int. J. Antenn. Propag..

[69] Zhang S., Whittow W., Seager R., Chauraya A., Vardaxoglou J.Y.C. (2017). Non-uniform mesh for embroidered microstrip antennas. IET Microw., Antennas Propag..

[70] Hertleer C., Rogier H., Vallozzi L., Van Langenhove L. (2009). A textile antenna for off-body communication integrated into protective clothing for firefighters. IEEE Trans. Antenn. Propag..

[71] Salvado R., Loss C., Gon, Pinho P. (2012). Textile materials for the design of wearable antennas: a survey. Sensors.

[72] AL-Haddad, Jamel M., Nursabirah, Nordin Anis (2021). Flexible antenna: a review of design, materials, fabrication, and applications. J. Phys. Conf..

[73] Kubo M., Li X., Kim C., Hashimoto M., Wiley B.J., Ham D., Whitesides G.M. (2010). Stretchable microfluidic radiofrequency antennas. Adv. Mater..

[74] Xiao G.G., Zhang Z., Lang S., Tao Y. (2016).

[75] Wang Y. (2019). Flexible RFID tag metal antenna on paper based substrate by inkjet printing technology. Adv. Funct. Mater..

[76] Lakafosis V., Rida A., Vyas R., Yang L., Nikolaou S., Tentzeris M.M. (Sept. 2010). Progress towards the first wireless sensor networks consisting of inkjet-printed, paper-based RFID-enabled sensor tags. Proc. IEEE.

[77] Yang L., Rida A., Vyas R., Tentzeris M.M. (2007). RFID tag and RF structures on a paper substrate using inkjet-printing technology. IEEE Trans. Microw. Theor. Tech..

[78] Liu Y., Xu L., Li Y., Ye T.T. (May 2019).

[79] Sindhu B., Kothuru A., Sahatiya P., Goel S., Nandi S. (July 2021). Laser-induced graphene printed wearable flexible antenna-based strain sensor for wireless human motion monitoring. IEEE Trans. Electron. Dev..

[80] Esakkimuthu Manikandan (2017).

[81] Suikkola J., Björninen T., Mosallaei M. (2016). Screen-printing fabrication and characterization of stretchable electronics. Sci. Rep..

[82] De Vita G., lannaccone G. (2004). Design criteria for the RF section of long range passive RFID systems. Proceedings Norchip Conference.

[83] Liu X., Lu W., Ye L., Li F., Zou D. (2017). Joint resource allocation of spectrum sensing and energy harvesting in an energy-harvesting-based cognitive sensor network. Sensors.

[84] Yan H., Montero J.G.M., Akhnoukh A., de Vreede L.C.N., Burghart J.N. (2005). Presented at the 8th Annu. Workshop Semiconductor Advances Future Electron. Sensors.

[85] Razavi B. (1997).

[86] Merz C., Kupris G., Niedernhuber M. (2014). 2014 International Conference on Applied Electronics.

[87] Munir B., Dyo V. (2018). On the impact of mobility on battery-less RF energy harvesting system performance. Sensors.

[88] Khaleel A.D. (2015).

[89] Tawde P.P. (2015). Half wave dipole antenna for satellite communication application. IJARSE.

